# YB-1 as an Oncoprotein: Functions, Regulation, Post-Translational Modifications, and Targeted Therapy

**DOI:** 10.3390/cells11071217

**Published:** 2022-04-04

**Authors:** Qiyan Yin, Min Zheng, Qianmei Luo, Dewei Jiang, Huifeng Zhang, Ceshi Chen

**Affiliations:** 1 Medical Faculty, Kunming University of Science and Technology, Kunming 650504, China; qiyanyin123@163.com; 2 Key Laboratory of Animal Models and Human Disease Mechanisms of Chinese Academy of Sciences and Yunnan Province, Kunming Institute of Zoology, Kunming 650201, China; zhengmin16255@163.com (M.Z.); 15752813580@163.com (Q.L.); jiangdewei@mail.kiz.ac.cn (D.J.); 3 Kunming College of Life Sciences, The University of the Chinese Academy of Sciences, Kunming 650201, China; 4 Department of Clinical Pharmacy, The First People’s Hospital of Yunnan Province/The Affiliated Hospital of Kunming University of Science and Technology, Kunming 650032, China; 5 Academy of Biomedical Engineering, Kunming Medical University, Kunming 650500, China; 6 The Third Affiliated Hospital, Kunming Medical University, Kunming 650118, China

**Keywords:** YB-1, transcription factor, cancer, RNA binding protein, therapeutic target

## Abstract

Y box binding protein 1 (YB-1) is a protein with a highly conserved cold shock domain (CSD) that also belongs to the family of DNA- and RNA-binding proteins. YB-1 is present in both the nucleus and cytoplasm and plays versatile roles in gene transcription, RNA splicing, DNA damage repair, cell cycle progression, and immunity. Cumulative evidence suggests that YB-1 promotes the progression of multiple tumor types and serves as a potential tumor biomarker and therapeutic target. This review comprehensively summarizes the emerging functions, mechanisms, and regulation of YB-1 in cancers, and further discusses targeted strategies.

## 1. Introduction

Y-box binding proteins (YB proteins) belong to the cold shock domain-containing protein family [1]. YB proteins include three members, YB-1, YB-2, and YB-3 [2,3]. They are highly conserved with respect to their cold shock domain (CSD) but contain very different C-terminal domain (CTD) sequences. Among the three members of the family, the protein sequences of CSDs share more than 90% identity, especially between YB-1 and YB-3, which were highly conserved and their CTDs are close in amino acid composition (Arg, Pro, Glu, Gln, and Gly amount to approximately 60% of all residues) and contain a large number of charged residues. In contrast, the N-terminal domains of the three YB proteins are the least homologous, although all are rich in alanine and proline residues [4].

The expression of YB proteins can be divided into somatic and germ cell-specific expression [5]. YB-1 is believed to play an important role in basic cellular functions, especially during the early stages of ontogenesis [6]. Germ-cell-specific YB-2 is abundant in growing oocytes and is essentially degraded during the late 2-cell stage [7]. YB-3 is highly expressed in mammalian embryos, and they are expressed in several tissues after birth, such as the heart, skeletal muscles, testicles, and blood vessels [6,8,9]. In the nucleus, YB proteins bind DNA sequences called Y-boxes (5′-CTGATTGGC/TC/TAA-3′) located in the promoter regions of target genes, thus regulating their transcription. Among the YB proteins, YB-1 is the most widely studied one which plays important roles in DNA repair, cell proliferation, and differentiation. YB-1 is an oncoprotein and is overexpressed in different types of cancers, including breast cancer [10]. A number of studies have shown that in response to stress, YB-1 translocates from the cytoplasm to the nucleus and binds to the promoter of its target genes, which regulates stemness [11], multidrug resistance [12], cell cycle [13], etc. Additionally, YB-1 was mainly reported as RNA binding proteins, especially as new RNA 5-methylcytosine reader, to regulate mRNA transcription, splicing, packaging, stabilization, and translation [14,15]. Therefore, the expression levels of the YB-1 protein are closely correlated with multidrug resistance, recurrence, metastasis, and poor prognosis in cancer patients.

Several review articles have recently summarized the function of YB-1 in various cancers [16,17]. In this review, we focus on newly described functions of YB-1 in cancer, encompassing its functions in the immune system and autophagy, its upstream regulation, post-translational modifications, and potential targeted strategies.

## 2. Protein Structure of YB-1

**Figure 1:**
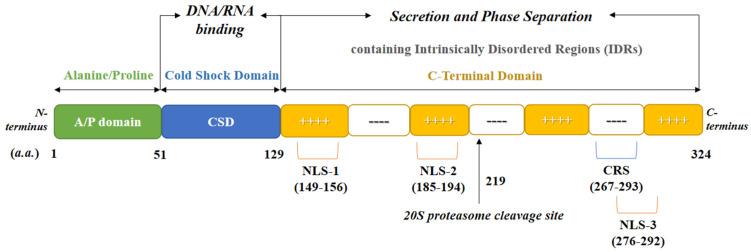
YB-1 protein structure and function domains. The YB-1 protein consists of 324 amino acid residues. YB-1 has an alanine and proline-rich A/P structural domain at the N-terminus, a cold-shock structural domain in the middle segment, and a C-terminal structural domain. The nuclear localization signal (NLS), S20 proteasome cleavage site, and cytoplasmic retention site (CRS) located at the C-terminus are also labeled.

YB-1 is a 324-amino acid protein with a predicted molecular weight of 35.9 KDa. YB-1 is a transcription and translation factor with multiple functions in the expression of various proteins. It consists of a highly conserved nucleic acid binding motif called CSD, an alanine/proline-rich N-terminal, and a C-terminal charged zipper characterized by alternating stretches of positively and negatively charged amino acids [18]. The protein domain structure of human YB-1 is shown in Figure 1.

Previous studies reported the solution structure of YB-1, where CSD is responsible for binding to nucleic acids [19]. A DNA binding domain consisting of positively charged and aromatic residues is present on the β-barrel surface of YB-1; however, the CSD binds weakly and without specificity to DNA [19]. The CSD consists of a closed five-stranded anti-parallel beta-barrel capped by a long flexible loop [14]. The latest study reported a high-resolution (1.7°) crystal structure of YB-1 CSD and different RNA oligomers, revealing the molecular basis of the interaction between YB-1 and RNA. These results show that CSD primarily interacts with RNA through π–π stacking interactions assembled by four highly conserved aromatic residues [20]. In addition, scientists adopted UV spectrophotometry, differential scanning calorimetry, and one-dimensional nuclear magnetic resonance hydrogen spectroscopy to study the properties of the YB-1 structure in depth and showed that CSD within the entire YB-1 chain has a well-packed tertiary structure [19].

## 3. The Functions and Mechanisms of YB-1

### 3.1. The Function of YB-1 in Cancers

As a multifunctional oncoprotein, YB-1 has been shown to be overexpressed and is often associated with poor prognoses in human cancers, such as breast cancer [21], ovarian cancer [22], liver cancer [23], lung cancer [24], colorectal cancer [25], prostate cancer [26], multiple myeloma [27], melanoma [28], osteosarcoma [29], glioblastoma [30], mesothelioma [31], and urothelial carcinoma of the bladder [32]. Cumulative evidence suggests that YB-1 regulates cancer cell behaviors, such as cell proliferation and cell cycle progression, stemness, migration and invasion, DNA damage repair (DDR), autophagy, tumor immunity, and multidrug resistance.

### 3.2. Cell Proliferation and Cell Cycle Progression

YB-1 promotes cell proliferation and cell cycle progression in multiple cancer cells. A reduction in YB-1 expression led to growth inhibition or apoptosis by downregulating downstream growth-promoting oncogenes [33]. YB-1 has been shown to activate multiple pro-proliferation genes, such as E2F transcription factor 1 (E2F1) [34], Cyclin A and Cyclin B1 [35], proliferating cell nuclear antigen (PCNA) [36], thymidine kinase 1 (TK1) [37], and epidermal growth factor receptor (EGFR) [38]. We recently reported that in basal-like breast cancer (BLBC) cells, YB-1 promotes KLF5 expression and cancer cell proliferation [39]. In addition, YB-1 transgenic mice develop different types of invasive breast cancer through genetic instability caused by mitotic failure and centrosome amplification [40]. YB-1 promotes the growth of other types of cancer cells, such as melanoma, adenocarcinoma, liver cancer, fibrosarcoma, colon cancer [41], lung cancer [42], prostate cancer [43], myeloma [44], acute myeloid leukemia [45], and spinal chordoma [46].

### 3.3. Cancer Stem-like Properties

Cancer stem cells (CSCs) comprise a small subpopulation of neoplastic cells within a tumor. CSCs possess the properties of self-renewal [47], differentiation, and indefinite proliferation, and they might be the primary cause of tumor initiation, progression, and recurrence in many cancers. In human breast cancer cell lines, YB-1 promotes cancer cell growth and drug resistance by binding the promoters of stem cell-related genes, including *CD44* and *CD49f* (integrin α6) [11]. Additionally, Mylona et al. reported the first clinicopathological study showing that YB-1 is an aggressive and “stem cell-like” tumor phenotypic trait [CD44^+^/CD24^−/low^] [48]. In breast cancer, the inhibition of P90 ribosomal S6 kinase (RSK), a key kinase involved in the phosphorylation of YB-1, eradicates the population of breast CSCs and overcomes drug resistance [49]. In addition, the inhibition of WAVE3 (WASF3), a protein involved in the nuclear translocation of YB-1, inhibits the expression of several CSC-related transcription factors, such as *NANOG*, *SOX2*, and *OCT4* [50]. In estrogen receptor (ER)-positive breast cancer, the activated ERα protein directly binds to the YB-1 promoter, promoting the expression of YB-1 and the formation of stem cells. YB-1 directly promotes Nanog transcription and the CSC properties of non-small cell lung cancer (NSCLC) cells [51]. In addition, in hepatocellular carcinoma (HCC) cells, YB-1 promotes Wnt/β-catenin signaling and induces stemness-related gene expression, maintaining the HCC-initiating cell population [52].

### 3.4. Invasion and Metastasis

Metastasis is a multistep process that mediates the distant spread of cancer cells from the primary tumor. The increased expression of YB-1 promotes migration and invasion in breast cancer [53]. YB-1 is highly expressed in approximately 70% of BLBC [54]. Another study found that the stable knockdown of YB-1 in the triple-negative breast cancer (TNBC) cell line MDA-MB-231 led to a reduction in cell invasion. YB-1 promotes the expression of MMP1 and β-catenin, which are known to increase cell adhesion and cell–matrix interactions, respectively [55]. Likewise, YB-1 binds to the promoter of *CTPS1* and activates its transcription, promoting the migration and proliferation of TNBC cells [56]. In lung cancer, YB-1 activates the transcription of *Nanog*, ultimately leading to an increase in the number of metastatic cells and the formation of metastatic colonization in a new location [51]. In malignant pleural mesothelioma (MPM), YB-1 knockdown significantly reduces the migration and invasion of MPM cells, and miR-31 exhibits a tumor suppressive function in MPM by directly targeting YB-1 [57]. In gastric cancer, YB-1 is highly expressed in more invasive gastric cancer cell lines and promotes gastric cancer cell migration but not invasion [58]. In nasopharyngeal carcinoma (NPC) CNE1 cells, in response to TGF-β1 treatment, along with the upregulation of YB-1, increased the expression of N-cadherin and Vimentin, while significantly downregulating the expression of E-cadherin and promoting epithelial-to-mesenchymal transition (EMT) in NPC cells [59]. In melanoma, YB-1 promotes a highly invasive phenotype [60]. Additionally, YB-1 promotes the metastasis of liver cancer [61], gastric cancer [58], skin squamous cell carcinoma [62], spinal chordoma [46], ovarian cancer [63], prostate cancer [64], and lung adenocarcinoma [65]. Interestingly, one study showed that human YB-1 inhibited AKT-dependent oncogenic transformation in NIH3T3 [66].

### 3.5. DNA Damage Repair (DDR)

During cancer treatment, radiotherapy and chemotherapeutic drugs work by directly or indirectly, inducing DNA damage. Severe DNA damage can activate programmed cell death to eliminate cells with catastrophic mutations [67]. In mammals, several DNA repair pathways are responsible for removing DNA damage, ensuring the stability of the genome [68]. In nonmalignant cells, YB-1 is primarily localized in the cytoplasm and YB-1 is translocated from the cytoplasm to the nucleus in response to genotoxic stress [69]. YB-1 has a high affinity for damaged DNA in response to cisplatin [36,70]. YB-1 also exhibits endonucleolytic and exonucleolytic activities in vitro. In addition, YB-1 binds to the DNA repair proteins MSH2, DNA polymerase δ, Ku80, and WRN75. A recent study demonstrated that human endonuclease III (hNTH1) is a DNA repair protein and bifunctional DNA glycosylase that binds to YB-1 in the nucleus, and the hNTH1-YB-1 interface plays a role in the response to cisplatin in MCF7 cells [71]. YB-1 can be PARylated (Poly(ADP-ribosylation)) by PARP1 during the process of interacting with damaged DNA [72]. This post-translational modification of YB-1 likely contributes to the regulation of DNA repair.

### 3.6. Autophagy

Autophagy is a highly conserved catabolic process of the intracellular degradation and recycling of biomacromolecules, subcellular structures and damaged organelles. A large number of studies have shown a strong relationship between autophagy and tumors. Autophagy can inhibit tumor growth in cancer, but paradoxically, the survival of some tumors depends on autophagy [73,74]. The overexpression of YB-1 promotes the P110β/Vps34/Beclin1 pathway to induce autophagy in NSCLC and decreases the sensitivity of NSCLC cells to cisplatin [75]. LINC00857 binds to the YB-1 protein, preventing its proteasome degradation and increasing its nuclear translocation. Subsequently, YB-1 binds to the promoter of MET to promote its transcription, promoting the anti-apoptotic and anti-autophagic properties of lung cancer cells [76]. In hepatic progenitor cells (HPCs) and liver fibrogenesis, TGF-β induces the nuclear translocation of YB-1 and promotes the transcription of *Atg7* which is involved in HPC expansion and liver fibrosis [77].

### 3.7. Tumor Immunity

The immune system functions in immune surveillance. When tumor cells invade, the immune system recognizes and eliminates them based on the tumor antigens expressed on their surface. However, in some cases, tumor cells evade the body’s immune surveillance through various mechanisms, proliferate malignantly, and form tumors. Studies have shown that the upregulation of YB-1 drives immune escape. For example, in chemotherapy-resistant HCC cells, YB-1 binds to the *PD-L1* promoter to upregulate its expression and decreases the secretion of the chemokines IL1β, IL10, and TGF-β in vitro. YB-1 knockdown blocks PD-L1 expression via T-cell activation in the tumor microenvironment, reversing resistance to chemotherapy [78]. The function of YB-1 in regulating tumor immunity has cut a conspicuous figure, so the combination therapy targeting YB-1 and immunotherapy is promising.

### 3.8. Multidrug Resistance

Chemotherapy is a universal and effective treatment for cancer. However, chemotherapy resistance inevitably occurs, and this phenotype is primarily due to the overexpression of the multidrug transporter P-glycoprotein [79], which is encoded by the multidrug resistance (*MDR1*) gene. YB-1 has been shown to induce MDR1 expression [80]. YB-1 is primarily localized in the cytoplasm, but when exposed to UV irradiation or anticancer agents, it translocates into the nucleus [81]. Nuclear YB-1 increases the expression of MDR1 in breast cancer cells, which provides a molecular mechanism of intrinsic multidrug resistance in breast cancer [21]. Feng et al. found that the long noncoding RNA MIR200CHG directly binds YB-1 and inhibits the degradation of the YB-1 protein through the ubiquitin–proteasome pathway [82]. In addition, MIR200CHG promotes YB-1 phosphorylation at serine 102 and nuclear translocation, promoting the proliferation, metastasis, and resistance to cisplatin of breast cancer. A subsequent series of studies further confirmed the association between YB-1 and drug resistance in different breast cancer cell lines and poor prognosis in patients [83,84,85]. Moreover, YB-1 increases the protein levels of EphA2 by inhibiting its proteasomal degradation, promoting RTK inhibitor sunitinib resistance in clear cell renal cell carcinoma [86]. YB-1 has also been implicated in the multidrug resistance of other cancers, such as osteosarcoma [29], synovial sarcoma [87], prostate cancer [26], and myeloma [27]. 

### 3.9. YB-1 as an RNA 5-Methylcytosine (m5C)-Binding Protein

YB-1 is newly defined as an RNA m5C binding protein (reader) [32]. Base modification is highly enriched in RNAs, and methylation is the most abundant form of RNA modification. 5-Methylcytosine (m5C) is one of the most predominant RNA methylation modifications and has been observed in RNAs since the 1970s. Recent studies have shown that YB-1 preferentially binds to m5C RNA through interaction with two tryptophan residues (Trp45 and Trp65) in the CSD of YB-119 [32]. Compared to normal tissues, the overexpression of the m5C methyltransferase NSUN2 and the m5C binding protein YB-1 in bladder cancer promotes the stability of *hepatoma-derived growth factor (HDGF)* mRNA in an m5C-dependent manner and ultimately promotes the proliferation and metastasis of bladder cancer [32]. We recently reported in BLBC cells that YB-1 also recognizes and stabilizes m5C-modified *KLF5* mRNA to promote cancer cell proliferation [39].

Consistently, YB-1 preferentially recognizes m5C-modified mRNAs which play essential roles in maternal mRNA stability and early embryogenesis in zebrafish together with the mRNA stabilizer poly(A)-binding protein cytoplasmic 1a (Pabpc1a) [88]. It has also been found that m5C-modified maternal mRNAs display higher stability than nonm5C-modified mRNAs during the maternal-to-zygotic transition (MZT) [88]. Recent studies have shown that m5C RNA modification plays an important role in the development of adult stem cells. Ypsilon Schachter (YPS), a homolog of human YB-1, was demonstrated to promote germline stem cell (GSC) maintenance, proliferation, and progeny differentiation in *Drosophila* ovary by preferentially binding to m5C-containing RNAs. Furthermore, human YB-1 can functionally replace YPS to support normal GSC development [89]. In addition, as an RNA-binding protein (RBP), YB-1 directly activates the cap-independent translation of mRNA encoding *Snail1* and other transcription factors, which are involved in the downregulation of epithelial and activation of mesenchymal genes [90]. Similarly, YB-1 enhances HIF1α protein expression by directly binding to *HIF1α* mRNA to activate HIF1α translation in sarcoma cells [91]. There are potential YB-1 binding sites in both the 5′- and 3′-untranslated regions (UTRs) of *AURKA* mRNA, and YB-1 promotes the expression of the AURKA protein by directly and specifically binding to *AURKA* mRNA, thereby promoting the proliferation and migration of NPC cells [92]. Whether YB-1-mediated protein translation and RNA stability fully depend on its RNA m5C binding function requires further investigation.

### 3.10. Phase Separation

Liquid–liquid phase separation (LLPS) in cells refers to the compartmentalization and concentration of biomacromolecules such as proteins, nucleic acids, and lipids into different condensates. Liquid-like condensates may undergo changes in the state of matter for example to gel and solid states, which are essential to achieve their function [93]. LLPS is involved in a variety of biological processes, including gene transcription, DNA damage repair, tumorigenesis, and autophagy [94,95,96]. The intrinsically disordered regions (IDRs) are recognized as important for the formation of membraneless condensates. It is noticed that the CTD of YB-1 has typical IDRs (Figure 1) and the deletion of the CTD blocks the formation of YB-1 puncta. In addition, the deletion of the CTD from YB-1 impairs the binding of YB-1 protein to biomolecular condensates formed in cells. Finally, when all tyrosine residues in the YB-1 C-terminal disordered region were replaced with serine/alanine residues or replaced all arginine and lysine residues with glycine residue, the formation of YB-1 condensates were inhibited in cells. These results suggest that the YB-1 phase separation requires interactions between tyrosine- and arginine-rich motifs [97]. YB-1 is a component of the G3BPs-mediated phase separation particles and its roles in LLPS regulation could be regulated by physiological and pathological conditions.

### 3.11. YB-1 as a Secreted Protein 

A number of proteins can be extracellularly secreted and stimulating tumor cell proliferation and metastasis [98]. Growing evidence suggests that YB-1 can be secreted. In thylakoid cells and monocytes, YB-1 is secreted in response to inflammatory stimuli via a non-classical pattern similar to a macrophage migration inhibitory factor [99]. The addition of the purified YB-1 protein to various cells promoted cell proliferation and migration [99]. Macrophages can also actively secrete YB-1. When macrophages are stimulated with LPS, YB-1 can interact with *IL-6* mRNA and transport it outside the cell via YB-1-enriched vesicles, thereby maintaining intracellular *IL-6* mRNA levels [100]. In contrast, it has been shown that oxidative stress promotes the secretion of YB-1 and that the addition of purified YB-1 protein to cells has an anti-proliferative effect [101]. Melanoma cells can also actively secrete YB-1 via a calcium- and ATP-dependent non-classical secretory pathway, and extracellular YB-1 can stimulate the migration and invasion of melanoma cells [102]. In addition, it has been shown that YB-1 is secreted as a component of exosomes. Exosomes containing YB-1 can promote angiogenesis in gastric cancer cells by promoting the proliferation and migration of vascular endothelial cells [103]. Given the important role of YB-1 as a secreted protein, the role of YB-1 in the tumor microenvironment should be further explored. 

## 4. Upstream Regulation of YB-1

**Figure 2:**
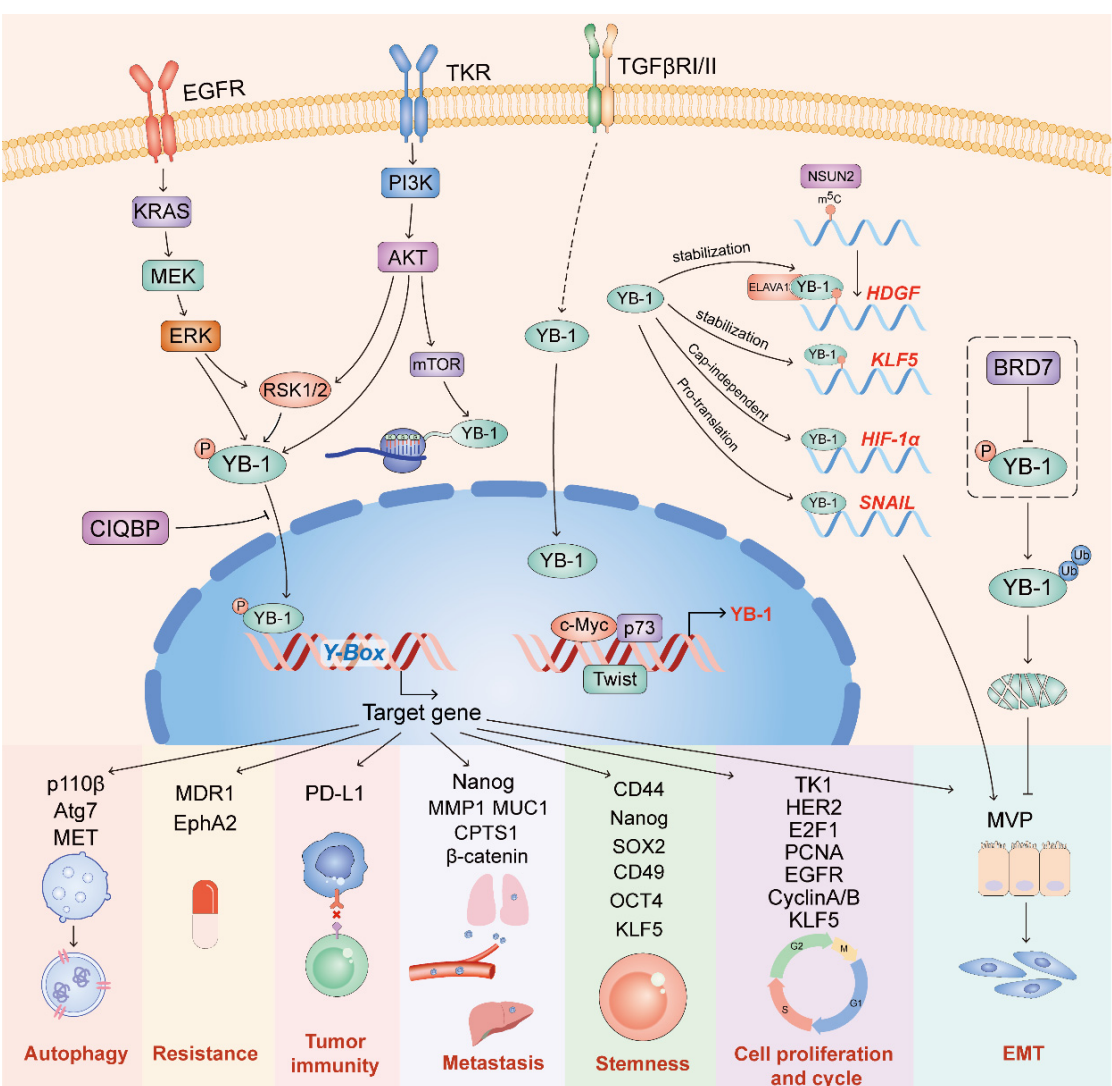
YB-1 is involved in multiple oncogenic signaling pathways. These pathways include PI3K/AKT, Ras/MEK, and TGF-β. YB-1 is phosphorylated by AKT, ERK, and RSK-2 into the nucleus to regulate the expression of target genes.

It is well known that activation mutations of the notorious signaling pathways PI3K/AKT/mTOR and Ras/MEK/ERK drive cell proliferation, migration, survival and drug resistance in a variety of cancers. The regulation of YB-1 involving these pathways is widely reported (Figure 2).

### 4.1. Upstream Regulators of YB-1

Previous studies have shown that both AKT and ERK promote YB-1′s phosphorylation and nuclear translocation [104,105]. Both AKT and ERK function via their downstream RSK, which in turn directly phosphorylates YB-1 [106]. In addition, YB-1 protein translation is controlled by mTOR, a downstream factor of PI3K/AKT [107]. Interestingly, *PIK3CA* transcription can be induced by YB-1 [108]. In the TGF-β signaling pathway, it has been suggested that TGF-β induces expression of YB-1 and its nuclear translocation, leading to EMT [109]. Consistently, YB-1 has been confirmed as a downstream target gene of Twist [43]. p73 stimulates the transcription of the YB-1 promoter by enhancing the recruitment of the c-Myc-Max complex to the E-box [110].

Accumulating evidence suggests that ΔNp63α interacts with YB-1, reduces YB-1 protein degradation, and promotes its nuclear accumulation, ultimately promoting keratin-forming cell proliferation [111]. The antimicrobial peptide LL-37 promotes the viability, migration, and invasion of skin squamous cell carcinoma [62] and malignant melanoma cells [112] by upregulating YB-1. The protein expression of YB-1 is negatively correlated with C1QBP expression in human renal cell carcinoma (RCC) clinical tissues, as shown by immunohistochemical staining. C1QBP interacts with YB-1, inhibits the phosphorylation of YB-1, and finally inhibits the invasion of RCC [113]. *BRD7* is a tumor-suppressor gene that inhibits breast cancer cell migration and invasion. *BRD7* can interact with YB-1, reducing the phosphorylation of YB-1 at S102, promoting its proteasomal degradation, and inhibiting breast tumor growth by suppressing EMT [114]. The distal-free homology cassette 4 (DLX4) is highly expressed in NPC cells, and DLX4 promotes NPC progression through the upregulation of YB-1 [115].

### 4.2. Multiple Non-Coding RNAs Regulate YB-1 in Cancer

**Figure 3:**
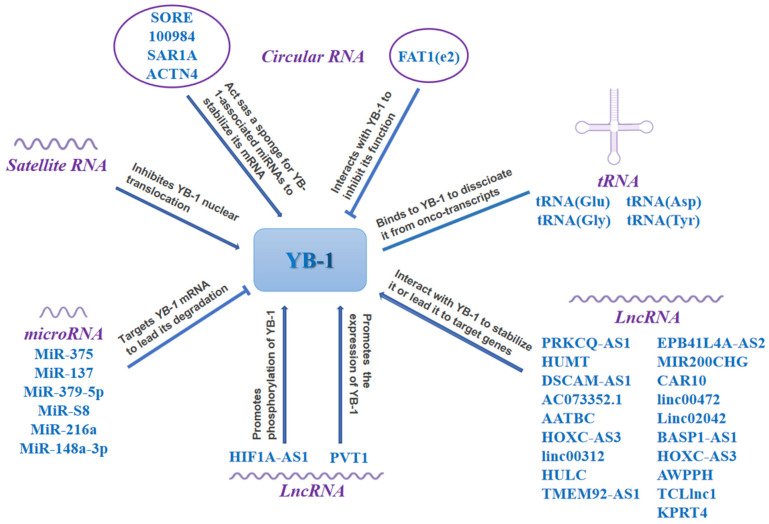
Multiple types of RNA regulate YB-1 expression or function. RNAs, including lncRNA, tRNA, microRNA, circular RNA, and satellite RNA, can regulate YB-1 expression at the protein/mRNA level, or influence YB-1 subcellular localization or phosphorylation. Picture resources are partly from https://www.figdraw.com/, accessed on 17 March 2022.

YB-1 is a well-known RBP. Recently, a number of noncoding RNAs, particularly lncRNAs and miRNAs, were reported to regulate YB-1 and tumorigenesis (Figure 3). 

#### 4.2.1. LncRNAs Regulate YB-1 in Cancer

LncRNAs are functionally defined as transcripts > 200 nt in length with no protein-coding potential and are uniquely expressed in differentiated tissues or specific cancer types [116]. Several lncRNAs have been reported to exhibit functions dependent on interacting with YB-1 in lung cancer. LncRNA CAR10 binds to and stabilizes YB-1 to upregulate the expression of EGFR and promotes lung cancer cell proliferation [117]. Linc00472 was also demonstrated to interact with YB-1 to regulate the EMT, inhibit cell stiffness and adhesion and ultimately suppress lung adenocarcinoma migration and invasion [118,119]. Linc00665 directly interacts with the YB-1 protein to promote its stabilization, leading to the nuclear accumulation of YB-1, acting as a transcription factor to activate the expression of angiopoietin-like protein 3/4 (ANGPTL3/4) to promote angiogenesis in lung adenocarcinoma [119]. In addition, Linc00312 promotes the migration of lung adenocarcinoma by directly binding to YB-1 to promote the formation of vasculogenic mimicry [120]. In breast cancer, the lncRNA HUMT forms a transcriptional complex with YB-1 and activates *FOXK1* transcription, which in turn increases the expression of VEGFC and ultimately promotes the progression of TNBC [121]. Similarly, lncRNA DSCAM-AS1 interacts with YB-1 and affects the recruitment of YB-1 into the *FOXA1* and *ERα* promoter regions, regulating their expression to promote breast cancer progression [122]. LncRNA AC073352.1 binds to and stabilizes YB-1 protein in breast cancer cells. Interestingly, exosomal lncRNA AC073352.1 also promote angiogenesis in HUVECs by binding to YB-1 [123]. LncRNA AATBC activates the YAP1/Hippo signaling pathway through the AATBC-YB-1-MST1 axis, promoting breast cancer migration and invasion [124]. LncRNA HOXC-AS3 promotes the transcriptional activation of *TK1* by binding to YB-1 to participate in breast carcinogenesis [125]. LncRNA MIR200CHG directly binds to YB-1 to inhibit its ubiquitination and degradation, simultaneously increasing YB-1 pS102 expression in the nucleus and cytoplasm to promote proliferation, invasion and drug resistance in breast cancer [82]. In addition, lncRNA HULC promotes the phosphorylation of YB-1 through the ERK pathway, which in turn regulates the interaction of YB-1 with certain oncogenic mRNAs and accelerates the translation of these mRNAs to promote liver cancer [126]. Interestingly, lncRNA HOXC-AS3 binds to YB-1 but has no effect on YB-1 expression. However, their interaction mediates gastric carcinogenesis through the transcriptional regulation of a large number of genes associated with cell proliferation and migration [127]. In pancreatic cancer, the lncRNA HIF1A-AS1 promotes the interaction of AKT with YB-1, inducing YB-1 phosphorylation. Additionally, HIF1A-AS1 recruits pYB-1 to the mRNA of *HIF1α*, promoting its translation, facilitating glycolysis and enhancing the resistance of pancreatic cancer cells to gemcitabine (a nucleoside antimetabolite) [128]. Likewise, lncRNAs that interact with YB-1 have been successively identified in liver cancer [129], gastric cancer [130], lymphoma [131], nasopharyngeal cancer [132], esophageal squamous cell carcinoma [133], melanoma [134], and clear cell renal cell carcinoma [135]. In addition, lncRNA PVT1 indirectly promotes YB-1 expression by sponging miR-216a-5p in colorectal cancer [136]. Similarly, lncRNA PRKCQ-AS1 indirectly upregulates the expression of YB-1 by competing with miR-1287-5p, leading to cell proliferation and migration in colorectal cancer [137]. Recently, our study identified a novel lncRNA KPRT4 that is directly transcriptionally regulated by KLF5, which promotes BLBC cell proliferation. Mechanistically, KPRT4 recruits the YB-1 transcription factor to the *KLF5* promoter by interacting with YB-1, thereby enhancing KLF5 transcription and ultimately establishing a feedforward circuit [138].

#### 4.2.2. MicroRNAs (miRNAs) Target YB-1 in Cancer

MiRNAs are a class of endogenous noncoding RNAs in eukaryotes that function to regulate gene expression and are approximately 20–25 nt in length. Their mechanism of action occurs primarily through binding to mRNA, leading to the degradation of mRNA and thus exerts gene silencing or translation inhibition [139]. MiR-375 directly binds to the 3′-UTR of *YB-1* mRNA to reduce its expression in breast cancer MCF-7 cells [140]. Likewise, miR-S8 and miR-216a inhibit YB-1 expression in human melanoma and pancreatic cancer [136,141]. Similarly, YB-1 was also identified as a direct target of miR-379-5p in NPC cells and miR-137 in MPM [57,142]. In NSCLC, miR-148a-3p targets YB-1, and miR-148a-3p knockdown significantly enhances YB-1 expression and promotes cancer cell growth [143].

#### 4.2.3. CircRNAs Regulate YB-1 in Cancer

Circular RNA (circRNA) is another type of noncoding RNA [144]. Compared to linear ncRNAs, circRNAs are more stable due to their closed-loop structure and are not easily degraded. CircRNA-SORE binds to the YB-1 protein in the cytoplasm, blocks E3 ubiquitin ligase precursor mRNA processing factor 19 (PRP19)-mediated YB-1 ubiquitination and degradation, and ultimately promotes sorafenib resistance in hepatocellular carcinoma [145]. Circ_100984 indirectly promotes YB-1 expression and EMT by binding to miR-432-3p, promoting breast cancer progression [146]. Similarly, circ-SAR1A upregulates the expression of YB-1 by acting as a sponge of miR-382 to promote the growth and invasion of renal cell carcinoma cells [147]. Interestingly, a novel circFAT1(e2) interacts with YB-1 protein in the nucleus and inhibits gastric cancer progression [148]. Likewise, in intrahepatic cholangiocarcinoma, circACTN4 also interacts with YB-1 and co-initiates the transcription of the downstream target *FZD7*, promoting the progression of intrahepatic cholangiocarcinoma [149].

#### 4.2.4. Other RNAs Regulate YB-1 in Cancer

Satellite DNA is primarily located in the chromosome’s mitotic and perisomal regions and consists of large monomeric arrays of highly repetitive noncoding sequences. Previous evidence has shown that satellite DNA is silent [150]. However, recent studies have demonstrated that these regions are indeed actively transcribed [151]. Surprisingly, both endogenous tRNAs and satellite RNAs are associated with YB-1. It has been shown that under stress, tRNAs are cleaved to produce different classes of tRNA-derived fragments (tRFs) that replace the 30 UTRs of *YB-1*’s mRNA to repress the expression of *YB-1* mRNA [152]. In response to stress, satellite RNA prevents the nuclear translocation of YB-1 by interacting with YB-1 to reduce DNA damage repair function [153].

### 4.3. Post-Translational Modification of YB-1

**Figure 4:**
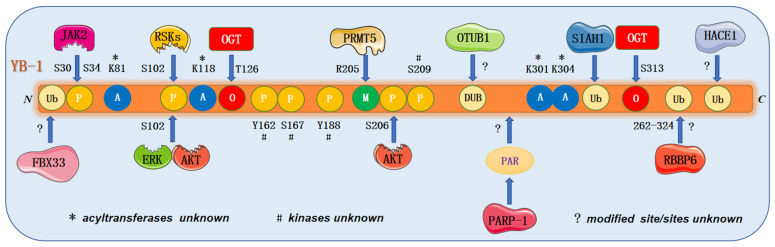
Post-translational modifications of the YB-1 protein. YB-1 is regulated by a variety of post-translational modifications, including phosphorylation (P), methylation (Me), acetylation (Ac), ubiquitination (Ub), O-glycosylation (OGT) and PARyation (PAR). The enzymes and sites modifying YB-1 are labeled.

The YB-1 protein undergoes multiple types of post-translational modification, including phosphorylation, ubiquitination, and acetylation. These modifications regulate the activity of the YB-1 protein and play important roles in tumorigenesis, metastasis, and multidrug resistance (Figure 4).

#### 4.3.1. Phosphorylation

Protein phosphorylation is catalyzed by protein kinases that transfer phosphate groups of ATP to serine, threonine, and tyrosine residues of target proteins. Phosphorylation at S102 in the CSD region is the most widely reported post-translational modification of YB-1 [104]. In addition, YB-1 phosphorylation sites contain S30/34 [154], S176 [155,156], S165 [157], Y162 [158,159,160], S209 [161], and Y188 [162]. First, AKT [161], ERK [163], and RSK1/2 [106] were identified as kinases that directly phosphorylate YB-1. Similarly, a number of factors that indirectly promote YB-1 phosphorylation were identified. LncRNA HULC and follicle-stimulating hormone (FSH) promote phosphorylation of YB-1 at S102 [163]. Additionally, integrin β8 promotes the phosphorylation of YB-1, leading to the activation of the NF-κB/BCL2 signaling pathway [164]. In JAK2-mutated myeloproliferative neoplasm cells, JAK2-dependent phosphorylation of YB-1 at S30/34 maintains its nuclear proportion and splicing function [154]. However, the tumor suppressor *BRD7* promotes YB-1 protein degradation by negatively regulating YB-1 phosphorylation at S102 [114]. Meanwhile, PDCD4 also reduces the protein levels of pYB-1 and blocks the nuclear translocation of YB-1, resulting in the inability of YB-1 to bind to the promoter of *MDR1* and reducing the resistance of cervical cancer to cisplatin [165].

#### 4.3.2. Acetylation

Protein acetylation is the process of adding acetyl groups to lysine residues (K) under the action of acetyltransferases. Limited studies reported that YB-1 can also be acetylated in pathological states or in cancers. In dialysis patients, the YB-1 K301/304 acetylation level is associated with systemic inflammation and vascular damage [166]. In cancer, the class I HDAC inhibitor MS-275 enhances YB-1 acetylation primarily on K81; in contrast, the YB-1 K81A mutant was MS-275-resistant and promoted the translational activation of NRF2, HIF1α, and G3BP1, promoting sarcoma metastasis [167]. 

#### 4.3.3. Methylation

Protein methylation sites primarily occur at two amino acid residues, arginine and lysine, where protein arginine methyltransferases catalyzes the transfer of methyl groups from S-adenosylmethionine to the guanidine nitrogen atom of arginine. PRMT5 is the major type II arginine methyltransferase, catalyzing the symmetric transfer of two methyl groups to arginine residues [168,169]. PRMT5 was reported to catalyze the methylation of YB-1 at R205, which is crucial for NF-κB activation and downstream target gene expression [170]. Interestingly, PRMT4 interacts with YB-1 to activate *VEGF* transcription to accelerate angiogenesis; however, no actual methylation of YB-1 was identified in the study [171].

#### 4.3.4. Ubiquitination and de-Ubiquitination

Ubiquitination is the process in which ubiquitin molecules are catalyzed by ubiquitin activating enzymes (E1), ubiquitin conjugation enzymes (E2), and ubiquitin ligases (E3) [172]. Five YB-1 E3 ubiquitin ligases, PRP19, retinoblastoma binding protein 6 (RBBP6), HECT domain and ankyrin repeat-containing ubiquitin ligase (HACE1), F-Box protein 33 (FBX33) and seven in absentia homolog 1 (SIAH1) were identified. First, circRNA-SORE binds to YB-1 protein in the cytoplasm and blocks YB-1 ubiquitination and degradation. PRP19 was identified as the first E3 that acts upon YB-1 by mass spectrometry [145]. An earlier study identified FBX33 as a component of the SCF E3 ubiquitin ligase, targeting YB-1 through its N-terminus for proteasome-dependent degradation [173]. RBBP6, an E3 ubiquitin ligase with a RING-finger structural domain, interacts with and ubiquitinates YB-1, leading to its proteasomal degradation [174]. Similarly, recent studies have shown that SIAH1 can ubiquitinate YB-1 at K304 and target it for proteasomal degradation, thereby reducing the resistance of epithelial ovarian cancer cells to cisplatin [175]. In contrast, HACE1 ubiquitinates YB-1 with noncanonical K27-linked polyubiquitin chains, which is required for the interaction of YB-1 with *tumor susceptibility gene 101* (*TSG101*), a key component of the ESCRT1 complex in the polycystic pathway; thus, TSG101 promotes YB-1 secretion to inhibit TGF-β-mediated EMT [176]. In addition, OTU domain-containing ubiquitin aldehyde-binding proteins Otubain1 (OTUB1) can interact with YB-1 as a deubiquitinating enzyme to reduce k48-linked YB-1 ubiquitination and thus stabilize YB-1. Activated protein C can reduce the damage caused by renal ischemia–reperfusion depending on the OTUB1/YB-1 interaction [177].

#### 4.3.5. O-glycosylation

O-glycosylation, a post-translational modification of serine and threonine groups on nuclear and cytoplasmic proteins with O-linked β-linked N-acetylglucosamine, is thought to modulate the function and activity of various intracellular proteins [178]. O-GlcNAc and phosphorylation were confirmed to coregulate YB-1’s function in promoting HCC proliferation. YB-1 and O-GlcNAc transferase (OGT) are highly expressed in HCC tissues, and four specific O-GlcNAc sites for YB-1, S32, T126, S209 and S313, were identified, in which T126 was the predominant site [179].

#### 4.3.6. PARylation

Poly(ADP-ribosylation) is primarily synthesized by poly(ADP-ribose) polymerases (PARPs). This post-translational modification is mainly related to DNA damage repair. We mentioned that YB-1 undergoes PARylation by PARP1. YB-1 PARylation reduces its affinity to DNA [72]. It has been shown that the CTDs of YB-1 can stimulate the activity of PARP1. YB-1 is highly expressed in chemotherapy-resistant tumors. The formation of a PARP1-YB-1-DNA ternary complex activates PARP1. The combination of DNA-damaging agents and PARP1 inhibitors may achieve better effects in tumor cells with high YB-1 expression [180,181].

### 4.4. Nuclear-Cytoplasmic Transport of YB-1

YB-1 exerts its functions in both the nucleus and cytoplasm, so its intracellular distribution must be strictly regulated. YB-1 has three nuclear localization signals (NLS) (149–156, 185–194, 276–292) [162] and a cytoplasmic retention signal (CRS) (247–267) [182] (Figure 1). It has been shown that the NLS sites of YB-1 are recognized by transportin-1 [183] and WAVE3 [50]. In nonmalignant cells, YB-1 is primarily located in the cytoplasm, but it accumulates in the nucleus in response to certain stimuli, such as UV radiation [81], hyperthermia [184], hypoxia [185], treatment with mitomycin C, cisplatin or doxorubicin [186,187], growth factors [188,189], and the cell cycle [35]. Under certain stress conditions, YB-1 undergoes specific proteolytic cleavage by the 20S proteasome, removing the CRS-containing portion of the YB-1 CTD, while the remaining NLS contains N-terminal fragments that accumulate in the nucleus [187,190]. In addition to proteasomal cleavage, YB-1 phosphorylation at S102 promotes its nuclear translocation [104]. It has also been reported that the nuclear accumulation of YB-1 requires a reduction in cytoplasmic mRNAs and YB-1 pS102 [191]. The conformational change caused by the dephosphorylation of YB-1 at S102, S165, and S176 facilitates the nuclear entry of YB-1 during late G2/M [192]. However, our research found that the RSK inhibitor-induced loss of YB-1 pS102 did not affect YB-1 subcellular localization in two BLBC cell lines [39]. Furthermore, it was reported that irradiation, TNF-α, EGF, or chemotherapeutics increased the YB-1 pS102 levels mediated by RSKs both in the cytoplasm and nucleus separately [193]. Instead, in hematopoietic cells, JAK2-mediated YB-1 pS30/S34 primarily determines its nuclear localization [154]. AKT S209 phosphorylation inhibits YB-1 nuclear translocation and prevents pS102-mediated YB-1 nuclear import. However, at present, little is known regarding the mechanism of YB-1 nuclear export. It has also been reported that the nuclear localization of YB-1 is hindered by elevated cytoplasmic mRNA levels [194], consistent with the loss of the DNA/RNA binding region disturbing its subcellular location. Whether the aforementioned protein modifications are also involved should be studied further. Collectively, YB-1 translocation could be highly pathophysiologically context- and cell type-dependent and remains to be further studied in the future.

## 5. Targeting YB-1 for Cancer Therapy

YB-1 is highly expressed in a variety of tumors, so it may serve as a diagnostic biomarker. A previous description of the upstream regulation and function of YB-1 suggests that YB-1 phosphorylation and nuclear accumulation typically indicate poor prognosis. A number of indirect inhibitors against YB-1 function have been developed and tested for cancer treatment in recent years (Table 1). Luteolin, an RSK inhibitor that inhibits Notch4 signaling by blocking YB-1 activation, thereby inhibits the growth of human-derived primary TNBC cells and induces apoptosis [195]. The sesquiterpene lactone 6-O-angeloylplenolin inhibits the nuclear translocation of YB-1 in colon cancer cells, leading to the downregulation of MDR1 and ultimately reducing the resistance of colon cancer cells to vincristine [196]. Similarly, 2,4-dihydroxy-5-pyrimidinyl imidothiocarbamate (DPI) also inhibits the nuclear translocation of YB-1, suppressing breast cancer cell proliferation and metastasis and increasing the efficacy of Adriamycin [197]. Additionally, anthraquinones aloe-emodin (AE) inhibits the expression of YB-1 by downregulating its ILK/Akt/mTOR signaling pathway, leading to the downregulation of the HER-2 expression and ultimately suppressing breast tumor metastasis and stemness [198]. The class I HDAC inhibitor MS-275 enhances the acetylation of YB-1 at K81, increasing the translation and synthesis of NRF2 by increasing its binding to the 3′-UTRs of *NRF2* and leading to a decrease in reactive oxygen species (ROS) in cells [167]. In HCC, 7-hydroxyisatin effectively inhibits actinomycin D-induced YB-1 nuclear translocation and inhibits expression of the YB-1 target genes *MDR1* and *EGFR*, thereby increasing the sensitivity of HepG2 cells to actinomycin D [199]. Recently, the first direct YB-1 inhibitor, an azopodophyllotoxin small molecule called SU056, was reported to biophysically bind to YB-1 and inhibit its expression, leading to cell cycle arrest and apoptosis in ovarian cancer cells [63]. Interestingly, HSc025, a small molecule compound that inhibits type I collagen production in fibroblasts, was identified to promote YB-1 entry into the nucleus, thereby regulating antagonistic TGF-β/Smad3 signaling in collagen gene expression, which could ultimately reduce fibrosis in the liver and kidney [200,201]. Meanwhile, RSK2 acts as an upstream factor and kinase of YB-1, and its inhibition decreases the phosphorylation and nucleation of YB-1. Currently reported inhibitors of RSKs include LJH685, LJI308, SL0101, BI-D1870, BIX 02565, and FMK [202].

## 6. Conclusions and Perspectives

As reviewed above, YB-1 is a critical transcription factor and RBP that promotes the transcription of target genes and regulates the stability and translation of mRNA. Functionally, YB-1 promotes the progression of a variety of tumors by regulating cell proliferation, autophagy, drug resistance, stemness, the tumor microenvironment, EMT, and metastasis. A variety of protein modifications of YB-1, such as phosphorylation, methylation, and ubiquitination also participate in its functional regulation. Mechanistically, YB-1 is regulated by a variety of signaling pathways, including PI3K/AKT/mTOR, Ras/MEK/ERK, and TGF-β. In addition, ncRNAs regulate YB-1 expression or function. A number of inhibitors have been discovered to block YB-1 nuclear translocation and suppress YB-1-mediated tumor progression. 

Accumulating evidence has heralded YB-1 as a potential therapeutic target for cancers; however, few studies have been conducted to develop small molecule inhibitors precisely targeting YB-1. Although recent studies have identified several small molecular compounds that inhibit the phosphorylation and nuclear translocation of YB-1, the exact mechanism remains unclear. Small molecule inhibitors and siRNA drugs that both directly and indirectly target YB-1 should be developed for cancer therapy. Given that YB-1 can regulate a large number of oncogenes, it is promising to target YB-1 in combination with other therapeutic modalities, such as immune checkpoint inhibitors EGFR inhibitors and anti-angiogenic drugs. Finally, the simultaneous targeting of YB-1 and its upstream regulators such as kinases or epigenetic modifiers is also an option. There is also great potential for the study of YB-1 as a secreted protein. Together, the upstream regulators, downstream target genes, interacting proteins, and signaling pathways involved in YB-1 regulation and its function require further investigation and could be helpful to explore new anti-cancer therapeutics. 

Although the cellular functions of YB-1 have been well characterized, its physiological and pathological functions, especially those regulating tumor immunity, microenvironment and metabolism, have not been explored. Therefore, there is an urgent need to develop tissue-specific YB-1 knockout and transgenic mouse models to study these functions. In conclusion, YB-1 is a potential target for cancer therapy, and further studies of its function and mechanism are warranted. Additional targeted therapies against YB-1 should be developed for preclinical and clinical studies.

## Figures and Tables

**Table 1:** Inhibitors of YB-1 pathway.

Inhibitors	Structure	Function and Mechanism	Disease	References
luteolin	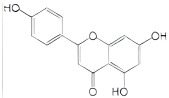	suppresses Notch4 signaling by blocking the activation of YB-1	TNBC	[195]
Sesquiterpene lactone 6-O-angeloylplenolin	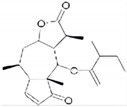	reverses vincristine resistance by inhibiting YB-1 nuclear translocation	colon carcinoma	[196]
Aloe-emodin	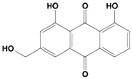	inhibits HER-2 expression through the downregulation of YB-1	HER-2 positive breast cancer	[198]
DPI	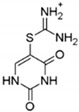	inhibits YB-1 nuclear translocation and increases the therapeutic potential of doxorubicin	breast cancer	[197]
MS-275	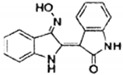	inhibits YB-1 deacetylation and reduces sarcoma metastasis	sarcoma	[167]
7-hydroxyindirubin	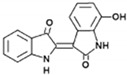	inhibits the actinomycin D-induced nuclear translocation of YB-1	HCC	[199]
SU056	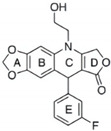	binds to YB-1 and inhibits its expression	ovarian cancer	[63]
HSc025	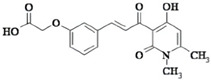	promotes YB-1 entry into the nucleus and reduces fibrosis in the liver and kidney	hepatic fibrosis and renal fibrosis	[200,201]

## References

[1] Spitkovsky D.D., Royer-Pokora B., Delius H., Kisseljov F., Jenkins N.A., Gilbert D.J., Copeland N.G., Royer H.D. (1992). Tissue restricted expression and chromosomal localization of the YB-1 gene encoding a 42 kD nuclear CCAAT binding protein. Nucleic Acids Res..

[2] Mastrangelo M.A., Kleene K.C. (2000). Developmental expression of Y-box protein 1 mRNA and alternatively spliced Y-box protein 3 mRNAs in spermatogenic cells in mice. Mol. Hum. Reprod..

[3] Sapru M.K., Gao J.P., Walke W., Burmeister M., Goldman D. (1996). Cloning and characterization of a novel transcriptional repressor of the nicotinic acetylcholine receptor delta-subunit gene. J. Biol. Chem..

[4] Mordovkina D., Lyabin D.N., Smolin E.A., Sogorina E.M., Ovchinnikov L.P., Eliseeva I. (2020). Y-Box Binding Proteins in mRNP Assembly, Translation, and Stability Control. Biomolecules.

[5] Gu W., Tekur S., Reinbold R., Eppig J.J., Choi Y.C., Zheng J.Z., Murray M.T., Hecht N.B. (1998). Mammalian male and female germ cells express a germ cell-specific Y-Box protein, MSY2. Biol. Reprod..

[6] Lu Z.H., Books J.T., Ley T.J. (2006). Cold shock domain family members YB-1 and MSY4 share essential functions during murine embryogenesis. Mol. Cell. Biol..

[7] Yu J., Hecht N.B., Schultz R.M. (2001). Expression of MSY2 in mouse oocytes and preimplantation embryos. Biol. Reprod..

[8] Davies H.G., Giorgini F., Fajardo M.A., Braun R.E. (2000). A sequence-specific RNA binding complex expressed in murine germ cells contains MSY2 and MSY4. Dev. Biol..

[9] Giorgini F., Davies H.G., Braun R.E. (2002). Translational repression by MSY4 inhibits spermatid differentiation in mice. Development.

[10] Ferreira A.R., Bettencourt M., Alho I., Costa A.L., Sousa A.R., Mansinho A., Abreu C., Pulido C., Macedo D., Vendrell I. (2017). Serum YB-1 (Y-box binding protein 1) as a biomarker of bone disease progression in patients with breast cancer and bone metastases. J. Bone Oncol..

[11] To K., Fotovati A., Reipas K.M., Law J.H., Hu K., Wang J., Astanehe A., Davies A.H., Lee L., Stratford A.L. (2010). Y-box binding protein-1 induces the expression of CD44 and CD49f leading to enhanced self-renewal, mammosphere growth, and drug resistance. Cancer Res..

[12] Kuwano M., Oda Y., Izumi H., Yang S.J., Uchiumi T., Iwamoto Y., Toi M., Fujii T., Yamana H., Kinoshita H. (2004). The role of nuclear Y-box binding protein 1 as a global marker in drug resistance. Mol. Cancer Ther..

[13] Harada M., Kotake Y., Ohhata T., Kitagawa K., Niida H., Matsuura S., Funai K., Sugimura H., Suda T., Kitagawa M. (2014). YB-1 promotes transcription of cyclin D1 in human non-small-cell lung cancers. Genes Cells.

[14] Kloks C.P., Spronk C.A., Lasonder E., Hoffmann A., Vuister G.W., Grzesiek S., Hilbers C.W. (2002). The solution structure and DNA-binding properties of the cold-shock domain of the human Y-box protein YB-1. J. Mol. Biol..

[15] Kleene K.C. (2018). Y-box proteins combine versatile cold shock domains and arginine-rich motifs (ARMs) for pleiotropic functions in RNA biology. Biochem. J..

[16] Alkrekshi A., Wang W., Rana P.S., Markovic V., Sossey-Alaoui K. (2021). A comprehensive review of the functions of YB-1 in cancer stemness, metastasis and drug resistance. Cell Signal..

[17] Sangermano F., Delicato A., Calabrò V. (2020). Y box binding protein 1 (YB-1) oncoprotein at the hub of DNA proliferation, damage and cancer progression. Biochimie.

[18] Wolffe A.P., Tafuri S., Ranjan M., Familari M. (1992). The Y-box factors: A family of nucleic acid binding proteins conserved from Escherichia coli to man. New Biol..

[19] Guryanov S.G., Filimonov V.V., Timchenko A.A., Melnik B.S., Kihara H., Kutyshenko V.P., Ovchinnikov L.P., Semisotnov G.V. (2013). The major mRNP protein YB-1: Structural and association properties in solution. Biochim. Biophys. Acta.

[20] Yang X.J., Zhu H., Mu S.R., Wei W.J., Yuan X., Wang M., Liu Y., Hui J., Huang Y. (2019). Crystal structure of a Y-box binding protein 1 (YB-1)-RNA complex reveals key features and residues interacting with RNA. J. Biol. Chem..

[21] Bargou R.C., Jürchott K., Wagener C., Bergmann S., Metzner S., Bommert K., Mapara M.Y., Winzer K.J., Dietel M., Dörken B. (1997). Nuclear localization and increased levels of transcription factor YB-1 in primary human breast cancers are associated with intrinsic MDR1 gene expression. Nat. Med..

[22] Kamura T., Yahata H., Amada S., Ogawa S., Sonoda T., Kobayashi H., Mitsumoto M., Kohno K., Kuwano M., Nakano H. (1999). Is nuclear expression of Y box-binding protein-1 a new prognostic factor in ovarian serous adenocarcinoma?. Cancer.

[23] Yasen M., Kajino K., Kano S., Tobita H., Yamamoto J., Uchiumi T., Kon S., Maeda M., Obulhasim G., Arii S. (2005). The up-regulation of Y-box binding proteins (DNA binding protein A and Y-box binding protein-1) as prognostic markers of hepatocellular carcinoma. Clin. Cancer Res..

[24] Shibahara K., Sugio K., Osaki T., Uchiumi T., Maehara Y., Kohno K., Yasumoto K., Sugimachi K., Kuwano M. (2001). Nuclear expression of the Y-box binding protein, YB-1, as a novel marker of disease progression in non-small cell lung cancer. Clin. Cancer Res..

[25] Shibao K., Takano H., Nakayama Y., Okazaki K., Nagata N., Izumi H., Uchiumi T., Kuwano M., Kohno K., Itoh H. (1999). Enhanced coexpression of YB-1 and DNA topoisomerase II alpha genes in human colorectal carcinomas. Int. J. Cancer.

[26] Giménez-Bonafé P., Fedoruk M.N., Whitmore T.G., Akbari M., Ralph J.L., Ettinger S., Gleave M.E., Nelson C.C. (2004). YB-1 is upregulated during prostate cancer tumor progression and increases P-glycoprotein activity. Prostate.

[27] Chatterjee M., Rancso C., Stuhmer T., Eckstein N., Andrulis M., Gerecke C., Lorentz H., Royer H.D., Bargou R.C. (2008). The Y-box binding protein YB-1 is associated with progressive disease and mediates survival and drug resistance in multiple myeloma. Blood.

[28] Schittek B., Psenner K., Sauer B., Meier F., Iftner T., Garbe C. (2007). The increased expression of Y box-binding protein 1 in melanoma stimulates proliferation and tumor invasion, antagonizes apoptosis and enhances chemoresistance. Int. J. Cancer.

[29] Oda Y., Sakamoto A., Shinohara N., Ohga T., Uchiumi T., Kohno K., Tsuneyoshi M., Kuwano M., Iwamoto Y. (1998). Nuclear expression of YB-1 protein correlates with P-glycoprotein expression in human osteosarcoma. Clin. Cancer Res..

[30] Faury D., Nantel A., Dunn S.E., Guiot M.C., Haque T., Hauser P., Garami M., Bognar L., Hanzely Z., Liberski P.P. (2007). Molecular profiling identifies prognostic subgroups of pediatric glioblastoma and shows increased YB-1 expression in tumors. J. Clin. Oncol..

[31] Goswami C.P., Nakshatri H. (2014). PROGgeneV2: Enhancements on the existing database. BMC Cancer.

[32] Chen X., Li A., Sun B.F., Yang Y., Han Y.N., Yuan X., Chen R.X., Wei W.S., Liu Y., Gao C.C. (2019). 5-methylcytosine promotes pathogenesis of bladder cancer through stabilizing mRNAs. Nat. Cell Biol..

[33] Lasham A., Print C.G., Woolley A.G., Dunn S.E., Braithwaite A.W. (2013). YB-1: Oncoprotein, prognostic marker and therapeutic target?. Biochem. J..

[34] Lasham A., Samuel W., Cao H., Patel R., Mehta R., Stern J.L., Reid G., Woolley A.G., Miller L.D., Black M.A. (2012). YB-1, the E2F pathway, and regulation of tumor cell growth. J. Natl. Cancer Inst..

[35] Jurchott K., Bergmann S., Stein U., Walther W., Janz M., Manni I., Piaggio G., Fietze E., Dietel M., Royer H.D. (2003). YB-1 as a cell cycle-regulated transcription factor facilitating cyclin A and cyclin B1 gene expression. J. Biol. Chem..

[36] Ise T., Nagatani G., Imamura T., Kato K., Takano H., Nomoto M., Izumi H., Ohmori H., Okamoto T., Ohga T. (1999). Transcription factor Y-box binding protein 1 binds preferentially to cisplatin-modified DNA and interacts with proliferating cell nuclear antigen. Cancer Res..

[37] Ladomery M., Sommerville J. (1995). A role for Y-box proteins in cell proliferation. BioEssays News Rev. Mol. Cell. Dev. Biol..

[38] Swamynathan S.K., Nambiar A., Guntaka R.V. (1998). Role of single-stranded DNA regions and Y-box proteins in transcriptional regulation of viral and cellular genes. FASEB J. Off. Publ. Fed. Am. Soc. Exp. Biol..

[39] Jiang D., Qiu T., Peng J., Li S., Tala, Ren W., Yang C., Wen Y., Chen C.H., Sun J. (2022). YB-1 is a positive regulator of KLF5 transcription factor in basal-like breast cancer. Cell Death Differ..

[40] Bergmann S., Royer-Pokora B., Fietze E., Jürchott K., Hildebrandt B., Trost D., Leenders F., Claude J.C., Theuring F., Bargou R. (2005). YB-1 provokes breast cancer through the induction of chromosomal instability that emerges from mitotic failure and centrosome amplification. Cancer Res..

[41] Swamynathan S.K., Varma B.R., Weber K.T., Guntaka R.V. (2002). Targeted disruption of one allele of the Y-box protein gene, Chk-YB-1b, in DT40 cells results in major defects in cell cycle. Biochem. Biophys. Res. Commun..

[42] Basaki Y., Taguchi K., Izumi H., Murakami Y., Kubo T., Hosoi F., Watari K., Nakano K., Kawaguchi H., Ohno S. (2010). Y-box binding protein-1 (YB-1) promotes cell cycle progression through CDC6-dependent pathway in human cancer cells. Eur. J. Cancer.

[43] Shiota M., Izumi H., Onitsuka T., Miyamoto N., Kashiwagi E., Kidani A., Yokomizo A., Naito S., Kohno K. (2008). Twist promotes tumor cell growth through YB-1 expression. Cancer Res..

[44] Cobbold L.C., Wilson L.A., Sawicka K., King H.A., Kondrashov A.V., Spriggs K.A., Bushell M., Willis A.E. (2010). Upregulated c-myc expression in multiple myeloma by internal ribosome entry results from increased interactions with and expression of PTB-1 and YB-1. Oncogene.

[45] Zhou H., Liu W., Zhou Y., Hong Z., Ni J., Zhang X., Li Z., Li M., He W., Zhang D. (2021). Therapeutic inhibition of GAS6-AS1/YBX1/MYC axis suppresses cell propagation and disease progression of acute myeloid leukemia. J. Exp. Clin. Cancer Res..

[46] Liang C., Ma Y., Yong L., Yang C., Wang P., Liu X., Zhu B., Zhou H., Liu X., Liu Z. (2019). Y-box binding protein-1 promotes tumorigenesis and progression via the epidermal growth factor receptor/AKT pathway in spinal chordoma. Cancer Sci..

[47] Koren S., Bentires-Alj M. (2015). Breast Tumor Heterogeneity: Source of Fitness, Hurdle for Therapy. Mol. Cell.

[48] Mylona E., Melissaris S., Giannopoulou I., Theohari I., Papadimitriou C., Keramopoulos A., Nakopoulou L. (2014). Y-box-binding protein 1 (YB1) in breast carcinomas: Relation to aggressive tumor phenotype and identification of patients at high risk for relapse. Eur. J. Surg. Oncol. J. Eur. Soc. Surg. Oncol. Br. Assoc. Surg. Oncol..

[49] Davies A.H., Reipas K., Hu K., Berns R., Firmino N., Stratford A.L., Dunn S.E. (2015). Inhibition of RSK with the novel small-molecule inhibitor LJI308 overcomes chemoresistance by eliminating cancer stem cells. Oncotarget.

[50] Bledzka K., Schiemann B., Schiemann W.P., Fox P., Plow E.F., Sossey-Alaoui K. (2017). The WAVE3-YB1 interaction regulates cancer stem cells activity in breast cancer. Oncotarget.

[51] Guo T., Kong J., Liu Y., Li Z., Xia J., Zhang Y., Zhao S., Li F., Li J., Gu C. (2017). Transcriptional activation of NANOG by YBX1 promotes lung cancer stem-like properties and metastasis. Biochem. Biophys. Res. Commun..

[52] Chao H.M., Huang H.X., Chang P.H., Tseng K.C., Miyajima A., Chern E. (2017). Y-box binding protein-1 promotes hepatocellular carcinoma-initiating cell progression and tumorigenesis via Wnt/β-catenin pathway. Oncotarget.

[53] Lim J.P., Shyamasundar S., Gunaratne J., Scully O.J., Matsumoto K., Bay B.H. (2017). YBX1 gene silencing inhibits migratory and invasive potential via CORO1C in breast cancer in vitro. BMC Cancer.

[54] Stratford A.L., Habibi G., Astanehe A., Jiang H., Hu K., Park E., Shadeo A., Buys T.P., Lam W., Pugh T. (2007). Epidermal growth factor receptor (EGFR) is transcriptionally induced by the Y-box binding protein-1 (YB-1) and can be inhibited with Iressa in basal-like breast cancer, providing a potential target for therapy. Breast Cancer Res. BCR.

[55] Lim J.P., Nair S., Shyamasundar S., Chua P.J., Muniasamy U., Matsumoto K., Gunaratne J., Bay B.H. (2019). Silencing Y-box binding protein-1 inhibits triple-negative breast cancer cell invasiveness via regulation of MMP1 and beta-catenin expression. Cancer Lett..

[56] Lin Y., Zhang J., Li Y., Guo W., Chen L., Chen M., Chen X., Zhang W., Jin X., Jiang M. (2022). CTPS1 promotes malignant progression of triple-negative breast cancer with transcriptional activation by YBX1. J. Transl. Med..

[57] Johnson T.G., Schelch K., Cheng Y.Y., Williams M., Sarun K.H., Kirschner M.B., Kao S., Linton A., Klebe S., McCaughan B.C. (2018). Dysregulated Expression of the MicroRNA miR-137 and Its Target YBX1 Contribute to the Invasive Characteristics of Malignant Pleural Mesothelioma. J. Thorac. Oncol..

[58] Guo T.T., Yu Y.N., Yip G.W., Matsumoto K., Bay B.H. (2013). Silencing the YB-1 gene inhibits cell migration in gastric cancer in vitro. Anat. Rec..

[59] Zhou L.L., Ni J., Feng W.T., Yao R., Yue S., Zhu Y.N., Tang H.Y., Lv L.Y., Feng J.F., Zhu W.G. (2017). High YBX1 expression indicates poor prognosis and promotes cell migration and invasion in nasopharyngeal carcinoma. Exp. Cell Res..

[60] Kosnopfel C., Sinnberg T., Sauer B., Busch C., Niessner H., Schmitt A., Forchhammer S., Grimmel C., Mertens P.R., Hailfinger S. (2018). YB-1 Expression and Phosphorylation Regulate Tumorigenicity and Invasiveness in Melanoma by Influencing EMT. Mol. Cancer Res..

[61] Wu Y., Yamada S., Izumi H., Li Z., Shimajiri S., Wang K.Y., Liu Y.P., Kohno K., Sasaguri Y. (2012). Strong YB-1 expression is associated with liver metastasis progression and predicts shorter disease-free survival in advanced gastric cancer. J. Surg. Oncol..

[62] Wang W., Zheng Y., Jia J., Li C., Duan Q., Li R., Wang X., Shao Y., Chen C., Yan H. (2017). Antimicrobial peptide LL-37 promotes the viability and invasion of skin squamous cell carcinoma by upregulating YB-1. Exp. Ther. Med..

[63] Tailor D., Resendez A., Garcia-Marques F.J., Pandrala M., Going C.C., Bermudez A., Kumar V., Rafat M., Nambiar D.K., Honkala A. (2021). Y box binding protein 1 inhibition as a targeted therapy for ovarian cancer. Cell Chem. Biol..

[64] Shiota M., Narita S., Habuchi T., Eto M. (2021). Validated prognostic significance of YB-1 genetic variation in metastatic prostate cancer. Pharm. J..

[65] Xie Q., Zhao S., Liu W., Cui Y., Li F., Li Z., Guo T., Yu W., Guo W., Deng W. (2021). YBX1 Enhances Metastasis and Stemness by Transcriptionally Regulating MUC1 in Lung Adenocarcinoma. Front. Oncol..

[66] Hohlfeld R., Brandt S., Bernhardt A., Gorny X., Schindele D., Jandrig B., Schostak M., Isermann B., Lindquist J.A., Mertens P.R. (2018). Crosstalk between Akt signaling and cold shock proteins in mediating invasive cell phenotypes. Oncotarget.

[67] Sancar A., Lindsey-Boltz L.A., Ünsal-Kaçmaz K., Linn S. (2004). Molecular mechanisms of mammalian DNA repair and the DNA damage checkpoints. Annu. Rev. Biochem..

[68] Tubbs A., Nussenzweig A. (2017). Endogenous DNA Damage as a Source of Genomic Instability in Cancer. Cell.

[69] Lasham A., Moloney S., Hale T., Homer C., Zhang Y.F., Murison J.G., Braithwaite A.W., Watson J. (2003). The Y-box-binding protein, YB1, is a potential negative regulator of the p53 tumor suppressor. J. Biol. Chem..

[70] Gaudreault I., Guay D., Lebel M. (2004). YB-1 promotes strand separation in vitro of duplex DNA containing either mispaired bases or cisplatin modifications, exhibits endonucleolytic activities and binds several DNA repair proteins. Nucleic Acids Res..

[71] Senarisoy M., Barette C., Lacroix F., De Bonis S., Stelter M., Hans F., Kleman J.P., Fauvarque M.O., Timmins J. (2020). Förster Resonance Energy Transfer Based Biosensor for Targeting the hNTH1-YB1 Interface as a Potential Anticancer Drug Target. ACS Chem. Biol..

[72] Alemasova E.E., Pestryakov P.E., Sukhanova M.V., Kretov D.A., Moor N.A., Curmi P.A., Ovchinnikov L.P., Lavrik O.I. (2015). Poly(ADP-ribosyl)ation as a new posttranslational modification of YB-1. Biochimie.

[73] Gozuacik D., Kimchi A. (2004). Autophagy as a cell death and tumor suppressor mechanism. Oncogene.

[74] Rosenfeldt M.T., Ryan K.M. (2009). The role of autophagy in tumour development and cancer therapy. Expert Rev. Mol. Med..

[75] Cui Y., Li F., Xie Q., Zhao S., Guo T., Guo P., Hu S., Hao J., Tian C., Yu W. (2020). YBX1 mediates autophagy by targeting p110β and decreasing the sensitivity to cisplatin in NSCLC. Cell Death Dis..

[76] Su W., Wang L., Zhao H., Hu S., Zhou Y., Guo C., Wu B., Li L., Yang Z., Beer D.G. (2020). LINC00857 Interacting with YBX1 to Regulate Apoptosis and Autophagy via MET and Phosphor-AMPKa Signaling. Mol. Ther. Nucleic Acids.

[77] Guo Y., Zhu J., Xu X., Shen B., Shen Z., Li B., Li F., Gu T., Cai X., Dong H. (2022). TGF-β/YB-1/Atg7 axis promotes the proliferation of hepatic progenitor cells and liver fibrogenesis. Biochim. Biophys. Acta Mol. Basis Dis..

[78] Tao Z., Ruan H., Sun L., Kuang D., Song Y., Wang Q., Wang T., Hao Y., Chen K. (2019). Targeting the YB-1/PD-L1 Axis to Enhance Chemotherapy and Antitumor Immunity. Cancer Immunol. Res..

[79] Gottesman M.M., Pastan I. (1993). Biochemistry of multidrug resistance mediated by the multidrug transporter. Annu. Rev. Biochem..

[80] Asakuno K., Kohno K., Uchiumi T., Kubo T., Sato S., Isono M., Kuwano M. (1994). Involvement of a DNA binding protein, MDR-NF1/YB-1, in human MDR1 gene expression by actinomycin D. Biochem. Biophys. Res. Commun..

[81] Koike K., Uchiumi T., Ohga T., Toh S., Wada M., Kohno K., Kuwano M. (1997). Nuclear translocation of the Y-box binding protein by ultraviolet irradiation. FEBS Lett..

[82] Tang L., Wei D., Xu X., Mao X., Mo D., Yan L., Xu W., Yan F. (2021). Long non-coding RNA MIR200CHG promotes breast cancer proliferation, invasion, and drug resistance by interacting with and stabilizing YB-1. NPJ Breast Cancer.

[83] Guay D., Evoy A.A., Paquet E., Garand C., Bachvarova M., Bachvarov D., Lebel M. (2008). The strand separation and nuclease activities associated with YB-1 are dispensable for cisplatin resistance but overexpression of YB-1 in MCF7 and MDA-MB-231 breast tumor cells generates several chemoresistance signatures. Int. J. Biochem. Cell Biol..

[84] Gluz O., Mengele K., Schmitt M., Kates R., Diallo-Danebrock R., Neff F., Royer H.D., Eckstein N., Mohrmann S., Ting E. (2009). Y-box-binding protein YB-1 identifies high-risk patients with primary breast cancer benefiting from rapidly cycled tandem high-dose adjuvant chemotherapy. J. Clin. Oncol..

[85] Fujita T., Ito K., Izumi H., Kimura M., Sano M., Nakagomi H., Maeno K., Hama Y., Shingu K., Tsuchiya S. (2005). Increased nuclear localization of transcription factor Y-box binding protein 1 accompanied by up-regulation of P-glycoprotein in breast cancer pretreated with paclitaxel. Clin. Cancer Res..

[86] Ruan H., Li S., Bao L., Zhang X. (2020). Enhanced YB1/EphA2 axis signaling promotes acquired resistance to sunitinib and metastatic potential in renal cell carcinoma. Oncogene.

[87] Oda Y., Ohishi Y., Saito T., Hinoshita E., Uchiumi T., Kinukawa N., Iwamoto Y., Kohno K., Kuwano M., Tsuneyoshi M. (2003). Nuclear expression of Y-box-binding protein-1 correlates with P-glycoprotein and topoisomerase II alpha expression, and with poor prognosis in synovial sarcoma. J. Pathol..

[88] Yang Y., Wang L., Han X., Yang W.L., Zhang M., Ma H.L., Sun B.F., Li A., Xia J., Chen J. (2019). RNA 5-Methylcytosine Facilitates the Maternal-to-Zygotic Transition by Preventing Maternal mRNA Decay. Mol. Cell.

[89] Zou F., Tu R., Duan B., Yang Z., Ping Z., Song X., Chen S., Price A., Li H., Scott A. (2020). Drosophila YBX1 homolog YPS promotes ovarian germ line stem cell development by preferentially recognizing 5-methylcytosine RNAs. Proc. Natl. Acad Sci. USA.

[90] Evdokimova V., Tognon C., Ng T., Ruzanov P., Melnyk N., Fink D., Sorokin A., Ovchinnikov L.P., Davicioni E., Triche T.J. (2009). Translational activation of snail1 and other developmentally regulated transcription factors by YB-1 promotes an epithelial-mesenchymal transition. Cancer Cell.

[91] El-Naggar A.M., Veinotte C.J., Cheng H., Grunewald T.G., Negri G.L., Somasekharan S.P., Corkery D.P., Tirode F., Mathers J., Khan D. (2015). Translational Activation of HIF1α by YB-1 Promotes Sarcoma Metastasis. Cancer Cell.

[92] Ban Y., Tan Y., Li X., Li X., Zeng Z., Xiong W., Li G., Xiang B., Yi M. (2021). RNA-binding protein YBX1 promotes cell proliferation and invasiveness of nasopharyngeal carcinoma cells via binding to AURKA mRNA. J. Cancer.

[93] Noda N.N., Wang Z., Zhang H. (2020). Liquid-liquid phase separation in autophagy. J. Cell Biol..

[94] Peng P.H., Hsu K.W., Wu K.J. (2021). Liquid-liquid phase separation (LLPS) in cellular physiology and tumor biology. Am. J. Cancer Res..

[95] Zhang H., Ji X., Li P., Liu C., Lou J., Wang Z., Wen W., Xiao Y., Zhang M., Zhu X. (2020). Liquid-liquid phase separation in biology: Mechanisms, physiological functions and human diseases. Sci. China. Life Sci..

[96] Alberti S., Gladfelter A., Mittag T. (2019). Considerations and Challenges in Studying Liquid-Liquid Phase Separation and Biomolecular Condensates. Cell.

[97] Liu X.M., Ma L., Schekman R. (2021). Selective sorting of microRNAs into exosomes by phase-separated YBX1 condensates. eLife.

[98] Paltridge J.L., Belle L., Khew-Goodall Y. (2013). The secretome in cancer progression. Biochim. Biophys. Acta.

[99] Frye B.C., Halfter S., Djudjaj S., Muehlenberg P., Weber S., Raffetseder U., En-Nia A., Knott H., Baron J.M., Dooley S. (2009). Y-box protein-1 is actively secreted through a non-classical pathway and acts as an extracellular mitogen. EMBO Rep..

[100] Kang S., Lee T.A., Ra E.A., Lee E., Choi H., Lee S., Park B. (2014). Differential control of interleukin-6 mRNA levels by cellular distribution of YB-1. PLoS ONE.

[101] Guarino A.M., Troiano A., Pizzo E., Bosso A., Vivo M., Pinto G., Amoresano A., Pollice A., La Mantia G., Calabrò V. (2018). Oxidative Stress Causes Enhanced Secretion of YB-1 Protein that Restrains Proliferation of Receiving Cells. Genes.

[102] Kosnopfel C., Sinnberg T., Sauer B., Niessner H., Muenchow A., Fehrenbacher B., Schaller M., Mertens P.R., Garbe C., Thakur B.K. (2020). Tumour Progression Stage-Dependent Secretion of YB-1 Stimulates Melanoma Cell Migration and Invasion. Cancers.

[103] Xue X., Huang J., Yu K., Chen X., He Y., Qi D., Wu Y. (2020). YB-1 transferred by gastric cancer exosomes promotes angiogenesis via enhancing the expression of angiogenic factors in vascular endothelial cells. BMC Cancer.

[104] Sutherland B.W., Kucab J., Wu J., Lee C., Cheang M.C., Yorida E., Turbin D., Dedhar S., Nelson C., Pollak M. (2005). Akt phosphorylates the Y-box binding protein 1 at Ser102 located in the cold shock domain and affects the anchorage-independent growth of breast cancer cells. Oncogene.

[105] Shen H., Xu W., Luo W., Zhou L., Yong W., Chen F., Wu C., Chen Q., Han X. (2011). Upregulation of mdr1 gene is related to activation of the MAPK/ERK signal transduction pathway and YB-1 nuclear translocation in B-cell lymphoma. Exp. Hematol..

[106] Stratford A.L., Fry C.J., Desilets C., Davies A.H., Cho Y.Y., Li Y., Dong Z., Berquin I.M., Roux P.P., Dunn S.E. (2008). Y-box binding protein-1 serine 102 is a downstream target of p90 ribosomal S6 kinase in basal-like breast cancer cells. Breast Cancer Res..

[107] Lyabin D.N., Eliseeva I.A., Ovchinnikov L.P. (2012). YB-1 synthesis is regulated by mTOR signaling pathway. PLoS ONE.

[108] Astanehe A., Finkbeiner M.R., Hojabrpour P., To K., Fotovati A., Shadeo A., Stratford A.L., Lam W.L., Berquin I.M., Duronio V. (2009). The transcriptional induction of PIK3CA in tumor cells is dependent on the oncoprotein Y-box binding protein-1. Oncogene.

[109] Ha B., Lee E.B., Cui J., Kim Y., Jang H.H. (2015). YB-1 overexpression promotes a TGF-β1-induced epithelial-mesenchymal transition via Akt activation. Biochem. Biophys. Res. Commun..

[110] Uramoto H., Izumi H., Ise T., Tada M., Uchiumi T., Kuwano M., Yasumoto K., Funa K., Kohno K. (2002). p73 Interacts with c-Myc to regulate Y-box-binding protein-1 expression. J. Biol. Chem..

[111] di Martino O., Troiano A., Guarino A.M., Pollice A., Vivo M., La Mantia G., Calabrò V. (2016). ΔNp63α controls YB-1 protein stability: Evidence on YB-1 as a new player in keratinocyte differentiation. Genes Cells.

[112] Jia J., Zheng Y., Wang W., Shao Y., Li Z., Wang Q., Wang Y., Yan H. (2017). Antimicrobial peptide LL-37 promotes YB-1 expression, and the viability, migration and invasion of malignant melanoma cells. Mol. Med. Rep..

[113] Yue D., Wang Y., Sun Y., Niu Y., Chang C. (2017). C1QBP Regulates YBX1 to Suppress the Androgen Receptor (AR)-Enhanced RCC Cell Invasion. Neoplasia.

[114] Niu W., Luo Y., Zhou Y., Li M., Wu C., Duan Y., Wang H., Fan S., Li Z., Xiong W. (2020). BRD7 suppresses invasion and metastasis in breast cancer by negatively regulating YB1-induced epithelial-mesenchymal transition. J. Exp. Clin. Cancer Res..

[115] Ling Z., Long X., Li J., Feng M. (2020). Homeodomain protein DLX4 facilitates nasopharyngeal carcinoma progression via up-regulation of YB-1. Genes Cells.

[116] Iyer M.K., Niknafs Y.S., Malik R., Singhal U., Sahu A., Hosono Y., Barrette T.R., Prensner J.R., Evans J.R., Zhao S. (2015). The landscape of long noncoding RNAs in the human transcriptome. Nat. Genet..

[117] Wei M.M., Zhou Y.C., Wen Z.S., Zhou B., Huang Y.C., Wang G.Z., Zhao X.C., Pan H.L., Qu L.W., Zhang J. (2016). Long non-coding RNA stabilizes the Y-box-binding protein 1 and regulates the epidermal growth factor receptor to promote lung carcinogenesis. Oncotarget.

[118] Deng X., Xiong W., Jiang X., Zhang S., Li Z., Zhou Y., Xiang B., Zhou M., Li X., Li G. (2020). LncRNA LINC00472 regulates cell stiffness and inhibits the migration and invasion of lung adenocarcinoma by binding to YBX1. Cell Death Dis..

[119] Cong Z., Diao Y., Li X., Jiang Z., Xu Y., Zhou H., Qiang Y., Wu H., Shen Y. (2020). Long non-coding RNA linc00665 interacts with YB-1 and promotes angiogenesis in lung adenocarcinoma. Biochem. Biophys. Res. Commun..

[120] Peng Z., Wang J., Shan B., Li B., Peng W., Dong Y., Shi W., Zhao W., He D., Duan M. (2018). The long noncoding RNA LINC00312 induces lung adenocarcinoma migration and vasculogenic mimicry through directly binding YBX1. Mol. Cancer.

[121] Zheng S., Yang L., Zou Y., Liang J.Y., Liu P., Gao G., Yang A., Tang H., Xie X. (2020). Long non-coding RNA HUMT hypomethylation promotes lymphangiogenesis and metastasis via activating FOXK1 transcription in triple-negative breast cancer. J. Hematol. Oncol..

[122] Zhang Y., Huang Y.X., Wang D.L., Yang B., Yan H.Y., Lin L.H., Li Y., Chen J., Xie L.M., Huang Y.S. (2020). LncRNA DSCAM-AS1 interacts with YBX1 to promote cancer progression by forming a positive feedback loop that activates FOXA1 transcription network. Theranostics.

[123] Kong X., Li J., Li Y., Duan W., Qi Q., Wang T., Yang Q., Du L., Mao H., Wang C. (2021). A novel long non-coding RNA AC073352.1 promotes metastasis and angiogenesis via interacting with YBX1 in breast cancer. Cell Death Dis..

[124] Wang M., Dai M., Wang D., Tang T., Xiong F., Xiang B., Zhou M., Li X., Li Y., Xiong W. (2021). The long noncoding RNA AATBC promotes breast cancer migration and invasion by interacting with YBX1 and activating the YAP1/Hippo signaling pathway. Cancer Lett..

[125] Su J., Yu B., Zhang C., Yi P., Li H., Xu C., Cao L., Chen P., Li M., Shen K. (2020). Long noncoding RNA HOXC-AS3 indicates a poor prognosis and regulates tumorigenesis by binding to YBX1 in breast cancer. Am. J. Transl. Res..

[126] Li D., Liu X., Zhou J., Hu J., Zhang D., Liu J., Qiao Y., Zhan Q. (2017). Long noncoding RNA HULC modulates the phosphorylation of YB-1 through serving as a scaffold of extracellular signal-regulated kinase and YB-1 to enhance hepatocarcinogenesis. Hepatology.

[127] Zhang E., He X., Zhang C., Su J., Lu X., Si X., Chen J., Yin D., Han L., De W. (2018). A novel long noncoding RNA HOXC-AS3 mediates tumorigenesis of gastric cancer by binding to YBX1. Genome Biol..

[128] Xu F., Huang M., Chen Q., Niu Y., Hu Y., Hu P., Chen D., He C., Huang K., Zeng Z. (2021). LncRNA HIF1A-AS1 Promotes Gemcitabine Resistance of Pancreatic Cancer by Enhancing Glycolysis through Modulating the AKT/YB1/HIF1α Pathway. Cancer Res..

[129] Zhao X., Liu Y., Yu S. (2017). Long noncoding RNA AWPPH promotes hepatocellular carcinoma progression through YBX1 and serves as a prognostic biomarker. Biochim. Biophys. Acta Mol. Basis Dis..

[130] Song S., He X., Wang J., Song H., Wang Y., Liu Y., Zhou Z., Yu Z., Miao D., Xue Y. (2021). A novel long noncoding RNA, TMEM92-AS1, promotes gastric cancer progression by binding to YBX1 to mediate CCL5. Mol. Oncol..

[131] Zhao P., Ji M.M., Fang Y., Li X., Yi H.M., Yan Z.X., Cheng S., Xu P.P., Janin A., Wang C.F. (2021). A novel lncRNA TCLlnc1 promotes peripheral T cell lymphoma progression through acting as a modular scaffold of HNRNPD and YBX1 complexes. Cell Death Dis..

[132] Du M., Hu X., Jiang X., Yin L., Chen J., Wen J., Fan Y., Peng F., Qian L., Wu J. (2021). LncRNA EPB41L4A-AS2 represses Nasopharyngeal Carcinoma Metastasis by binding to YBX1 in the Nucleus and Sponging MiR-107 in the Cytoplasm. Int. J. Biol. Sci..

[133] Du J., Zhang G., Qiu H., Yu H., Yuan W. (2020). A novel positive feedback loop of linc02042 and c-Myc mediated by YBX1 promotes tumorigenesis and metastasis in esophageal squamous cell carcinoma. Cancer Cell Int..

[134] Li Y., Gao Y., Niu X., Tang M., Li J., Song B., Guan X. (2021). LncRNA BASP1-AS1 interacts with YBX1 to regulate Notch transcription and drives the malignancy of melanoma. Cancer Sci..

[135] Zhao P., Deng Y., Wu Y., Guo Q., Zhou L., Yang X., Wang C. (2021). Long noncoding RNA SNHG6 promotes carcinogenesis by enhancing YBX1-mediated translation of HIF1α in clear cell renal cell carcinoma. FASEB J..

[136] Zeng X., Liu Y., Zhu H., Chen D., Hu W. (2019). Downregulation of miR-216a-5p by long noncoding RNA PVT1 suppresses colorectal cancer progression via modulation of YBX1 expression. Cancer Manag. Res..

[137] Cui G., Zhao H., Li L. (2020). Long noncoding RNA PRKCQ-AS1 promotes CRC cell proliferation and migration via modulating miR-1287-5p/YBX1 axis. J. Cell. Biochem..

[138] Du G., Sun J., Li Z., Zhang Q., Liu W., Yang C., Zhao P., Wang X., Yin Q., Luo Y. (2022). A feedforward circuit between KLF5 and lncRNA KPRT4 contributes to basal-like breast cancer. Cancer Lett..

[139] Vishnoi A., Rani S. (2017). MiRNA Biogenesis and Regulation of Diseases: An Overview. Methods Mol. Biol..

[140] Liu S.L., Sui Y.F., Lin M.Z. (2016). MiR-375 is epigenetically downregulated due to promoter methylation and modulates multi-drug resistance in breast cancer cells via targeting YBX1. Eur. Rev. Med. Pharmacol. Sci..

[141] Yang F., Wei J., Zhang S., Zhang X. (2017). Shrimp miR-S8 Suppresses the Stemness of Human Melanoma Stem-like Cells by Targeting the Transcription Factor YB-1. Cancer Res..

[142] Zhang F., Duan C., Yin S., Tian Y. (2020). MicroRNA-379-5p/YBX1 Axis Regulates Cellular EMT to Suppress Migration and Invasion of Nasopharyngeal Carcinoma Cells. Cancer Manag. Res..

[143] Su H., Fan G., Huang J., Qiu X. (2021). YBX1 regulated by Runx3-miR-148a-3p axis facilitates non-small-cell lung cancer progression. Cell Signal..

[144] Memczak S., Jens M., Elefsinioti A., Torti F., Krueger J., Rybak A., Maier L., Mackowiak S.D., Gregersen L.H., Munschauer M. (2013). Circular RNAs are a large class of animal RNAs with regulatory potency. Nature.

[145] Xu J., Ji L., Liang Y., Wan Z., Zheng W., Song X., Gorshkov K., Sun Q., Lin H., Zheng X. (2020). CircRNA-SORE mediates sorafenib resistance in hepatocellular carcinoma by stabilizing YBX1. Signal. Transduct. Target. Ther..

[146] Tong L., Yang H., Xiong W., Tang G., Zu X., Qi L. (2021). circ_100984-miR-432-3p axis regulated c-Jun/YBX-1/β-catenin feedback loop promotes bladder cancer progression. Cancer Sci..

[147] Zhao X., Zhao Z., Xu W., Liu H., Chang J., Xu W., Li S., Cao S., Hou J. (2020). Circ-SAR1A Promotes Renal Cell Carcinoma Progression Through miR-382/YBX1 Axis. Cancer Manag. Res..

[148] Fang J., Hong H., Xue X., Zhu X., Jiang L., Qin M., Liang H., Gao L. (2019). A novel circular RNA, circFAT1(e2), inhibits gastric cancer progression by targeting miR-548g in the cytoplasm and interacting with YBX1 in the nucleus. Cancer Lett..

[149] Chen Q., Wang H., Li Z., Li F., Liang L., Zou Y., Shen H., Li J., Xia Y., Cheng Z. (2022). Circular RNA ACTN4 promotes intrahepatic cholangiocarcinoma progression by recruiting YBX1 to initiate FZD7 transcription. J. Hepatol..

[150] Plohl M., Meštrović N., Mravinac B. (2014). Centromere identity from the DNA point of view. Chromosoma.

[151] Biscotti M.A., Canapa A., Forconi M., Olmo E., Barucca M. (2015). Transcription of tandemly repetitive DNA: Functional roles. Chromosome Res. Int. J. Mol. Supramol. Evol. Asp. Chromosome Biol..

[152] Goodarzi H., Liu X., Nguyen H.C., Zhang S., Fish L., Tavazoie S.F. (2015). Endogenous tRNA-Derived Fragments Suppress Breast Cancer Progression via YBX1 Displacement. Cell.

[153] Kishikawa T., Otsuka M., Yoshikawa T., Ohno M., Ijichi H., Koike K. (2016). Satellite RNAs promote pancreatic oncogenic processes via the dysfunction of YBX1. Nat. Commun..

[154] Jayavelu A.K., Schnöder T.M., Perner F., Herzog C., Meiler A., Krishnamoorthy G., Huber N., Mohr J., Edelmann-Stephan B., Austin R. (2020). Splicing factor YBX1 mediates persistence of JAK2-mutated neoplasms. Nature.

[155] Martin M., Hua L., Wang B., Wei H., Prabhu L., Hartley A.V., Jiang G., Liu Y., Lu T. (2017). Novel Serine 176 Phosphorylation of YBX1 Activates NF-κB in Colon Cancer. J. Biol. Chem..

[156] Sharma K., D’Souza R.C., Tyanova S., Schaab C., Wiśniewski J.R., Cox J., Mann M. (2014). Ultradeep human phosphoproteome reveals a distinct regulatory nature of Tyr and Ser/Thr-based signaling. Cell Rep..

[157] Prabhu L., Mundade R., Wang B., Wei H., Hartley A.V., Martin M., McElyea K., Temm C.J., Sandusky G., Liu Y. (2015). Critical role of phosphorylation of serine 165 of YBX1 on the activation of NF-κB in colon cancer. Oncotarget.

[158] Kasyapa C., Gu T.L., Nagarajan L., Polakiewicz R., Cowell J.K. (2009). Phosphorylation of the SSBP2 and ABL proteins by the ZNF198-FGFR1 fusion kinase seen in atypical myeloproliferative disorders as revealed by phosphopeptide-specific MS. Proteomics.

[159] Bennetzen M.V., Larsen D.H., Bunkenborg J., Bartek J., Lukas J., Andersen J.S. (2010). Site-specific phosphorylation dynamics of the nuclear proteome during the DNA damage response. Mol. Cell. Proteom. MCP.

[160] Kettenbach A.N., Schweppe D.K., Faherty B.K., Pechenick D., Pletnev A.A., Gerber S.A. (2011). Quantitative phosphoproteomics identifies substrates and functional modules of Aurora and Polo-like kinase activities in mitotic cells. Sci. Signal..

[161] Sogorina E.M., Kim E.R., Sorokin A.V., Lyabin D.N., Ovchinnikov L.P., Mordovkina D.A., Eliseeva I.A. (2021). YB-1 Phosphorylation at Serine 209 Inhibits Its Nuclear Translocation. Int. J. Mol. Sci..

[162] van Roeyen C.R., Scurt F.G., Brandt S., Kuhl V.A., Martinkus S., Djudjaj S., Raffetseder U., Royer H.D., Stefanidis I., Dunn S.E. (2013). Cold shock Y-box protein-1 proteolysis autoregulates its transcriptional activities. Cell Commun. Signal. CCS.

[163] Donaubauer E.M., Hunzicker-Dunn M.E. (2016). Extracellular Signal-regulated Kinase (ERK)-dependent Phosphorylation of Y-Box-binding Protein 1 (YB-1) Enhances Gene Expression in Granulosa Cells in Response to Follicle-stimulating Hormone (FSH). J. Biol. Chem..

[164] Liu S., Chen L., Zhao H., Li Q., Hu R., Wang H. (2020). Integrin β8 facilitates tumor growth and drug resistance through a Y-box binding protein 1-dependent signaling pathway in bladder cancer. Cancer Sci..

[165] Liu D., Ke J., Liu Y., Rao H., Tang Z., Liu Y., Zhang Z., You L., Luo X., Sun Z. (2021). The interaction between PDCD4 and YB1 is critical for cervical cancer stemness and cisplatin resistance. Mol. Carcinog..

[166] Ewert L., Fischer A., Brandt S., Scurt F.G., Philipsen L., Müller A.J., Girndt M., Zenclussen A.C., Lindquist J.A., Gorny X. (2018). Cold shock Y-box binding protein-1 acetylation status in monocytes is associated with systemic inflammation and vascular damage. Atherosclerosis.

[167] El-Naggar A.M., Somasekharan S.P., Wang Y., Cheng H., Negri G.L., Pan M., Wang X.Q., Delaidelli A., Rafn B., Cran J. (2019). Class I HDAC inhibitors enhance YB-1 acetylation and oxidative stress to block sarcoma metastasis. EMBO Rep..

[168] Xiao W., Chen X., Liu L., Shu Y., Zhang M., Zhong Y. (2019). Role of protein arginine methyltransferase 5 in human cancers. Biomed. Pharmacother. Biomed. Pharmacother..

[169] Zhang B., Zhang S., Zhu L., Chen X., Zhao Y., Chao L., Zhou J., Wang X., Zhang X., Ma N. (2017). Arginine methyltransferase inhibitor 1 inhibits gastric cancer by downregulating eIF4E and targeting PRMT5. Toxicol. Appl. Pharmacol..

[170] Hartley A.V., Wang B., Mundade R., Jiang G., Sun M., Wei H., Sun S., Liu Y., Lu T. (2020). PRMT5-mediated methylation of YBX1 regulates NF-κB activity in colorectal cancer. Sci. Rep..

[171] Yan S., Hu J., Li J., Wang P., Wang Y., Wang Z. (2021). PRMT4 drives post-ischemic angiogenesis via YB1/VEGF signaling. J. Mol. Med..

[172] Pickart C.M. (2001). Mechanisms underlying ubiquitination. Annu. Rev. Biochem..

[173] Lutz M., Wempe F., Bahr I., Zopf D., von Melchner H. (2006). Proteasomal degradation of the multifunctional regulator YB-1 is mediated by an F-Box protein induced during programmed cell death. FEBS Lett..

[174] Chibi M., Meyer M., Skepu A., DJ G.R., Moolman-Smook J.C., Pugh D.J. (2008). RBBP6 interacts with multifunctional protein YB-1 through its RING finger domain, leading to ubiquitination and proteosomal degradation of YB-1. J. Mol. Biol..

[175] Gao W., Chen L., Lin L., Yang M., Li T., Wei H., Sha C., Xing J., Zhang M., Zhao S. (2022). SIAH1 reverses chemoresistance in epithelial ovarian cancer via ubiquitination of YBX-1. Oncogenesis.

[176] Palicharla V.R., Maddika S. (2015). HACE1 mediated K27 ubiquitin linkage leads to YB-1 protein secretion. Cell Signal..

[177] Dong W., Wang H., Shahzad K., Bock F., Al-Dabet M.M., Ranjan S., Wolter J., Kohli S., Hoffmann J., Dhople V.M. (2015). Activated Protein C Ameliorates Renal Ischemia-Reperfusion Injury by Restricting Y-Box Binding Protein-1 Ubiquitination. J. Am. Soc. Nephrol. JASN.

[178] Comer F.I., Hart G.W. (2000). O-Glycosylation of nuclear and cytosolic proteins. Dynamic interplay between O-GlcNAc and O-phosphate. J. Biol. Chem..

[179] Liu Q., Tao T., Liu F., Ni R., Lu C., Shen A. (2016). Hyper-O-GlcNAcylation of YB-1 affects Ser102 phosphorylation and promotes cell proliferation in hepatocellular carcinoma. Exp. Cell Res..

[180] Alemasova E.E., Naumenko K.N., Kurgina T.A., Anarbaev R.O., Lavrik O.I. (2018). The multifunctional protein YB-1 potentiates PARP1 activity and decreases the efficiency of PARP1 inhibitors. Oncotarget.

[181] Naumenko K.N., Sukhanova M.V., Hamon L., Kurgina T.A., Alemasova E.E., Kutuzov M.M., Pastré D., Lavrik O.I. (2020). Regulation of Poly(ADP-Ribose) Polymerase 1 Activity by Y-Box-Binding Protein 1. Biomolecules.

[182] Woolley A.G., Algie M., Samuel W., Harfoot R., Wiles A., Hung N.A., Tan P.H., Hains P., Valova V.A., Huschtscha L. (2011). Prognostic association of YB-1 expression in breast cancers: A matter of antibody. PLoS ONE.

[183] Mordovkina D.A., Kim E.R., Buldakov I.A., Sorokin A.V., Eliseeva I.A., Lyabin D.N., Ovchinnikov L.P. (2016). Transportin-1-dependent YB-1 nuclear import. Biochem. Biophys. Res. Commun..

[184] Stein U., Jürchott K., Walther W., Bergmann S., Schlag P.M., Royer H.D. (2001). Hyperthermia-induced nuclear translocation of transcription factor YB-1 leads to enhanced expression of multidrug resistance-related ABC transporters. J. Biol. Chem..

[185] Rauen T., Frye B.C., Wang J., Raffetseder U., Alidousty C., En-Nia A., Floege J., Mertens P.R. (2016). Cold shock protein YB-1 is involved in hypoxia-dependent gene transcription. Biochem. Biophys. Res. Commun..

[186] Ohga T., Koike K., Ono M., Makino Y., Itagaki Y., Tanimoto M., Kuwano M., Kohno K. (1996). Role of the human Y box-binding protein YB-1 in cellular sensitivity to the DNA-damaging agents cisplatin, mitomycin C, and ultraviolet light. Cancer Res..

[187] Kim E.R., Selyutina A.A., Buldakov I.A., Evdokimova V., Ovchinnikov L.P., Sorokin A.V. (2013). The proteolytic YB-1 fragment interacts with DNA repair machinery and enhances survival during DNA damaging stress. Cell Cycle.

[188] Basaki Y., Hosoi F., Oda Y., Fotovati A., Maruyama Y., Oie S., Ono M., Izumi H., Kohno K., Sakai K. (2007). Akt-dependent nuclear localization of Y-box-binding protein 1 in acquisition of malignant characteristics by human ovarian cancer cells. Oncogene.

[189] Higashi K., Inagaki Y., Suzuki N., Mitsui S., Mauviel A., Kaneko H., Nakatsuka I. (2003). Y-box-binding protein YB-1 mediates transcriptional repression of human alpha 2(I) collagen gene expression by interferon-gamma. J. Biol. Chem..

[190] Sorokin A.V., Selyutina A.A., Skabkin M.A., Guryanov S.G., Nazimov I.V., Richard C., Th’ng J., Yau J., Sorensen P.H., Ovchinnikov L.P. (2005). Proteasome-mediated cleavage of the Y-box-binding protein 1 is linked to DNA-damage stress response. EMBO J..

[191] Kretov D.A., Mordovkina D.A., Eliseeva I.A., Lyabin D.N., Polyakov D.N., Joshi V., Desforges B., Hamon L., Lavrik O.I., Pastré D. (2019). Inhibition of Transcription Induces Phosphorylation of YB-1 at Ser102 and Its Accumulation in the Nucleus. Cells.

[192] Mehta S., McKinney C., Algie M., Verma C.S., Kannan S., Harfoot R., Bartolec T.K., Bhatia P., Fisher A.J., Gould M.L. (2020). Dephosphorylation of YB-1 is Required for Nuclear Localisation During G(2) Phase of the Cell Cycle. Cancers.

[193] Tiwari A., Rebholz S., Maier E., Dehghan Harati M., Zips D., Sers C., Rodemann H.P., Toulany M. (2018). Stress-Induced Phosphorylation of Nuclear YB-1 Depends on Nuclear Trafficking of p90 Ribosomal S6 Kinase. Int. J. Mol. Sci..

[194] Tanaka T., Kasai M., Kobayashi S. (2018). Mechanism responsible for inhibitory effect of indirubin 3’-oxime on anticancer agent-induced YB-1 nuclear translocation in HepG2 human hepatocellular carcinoma cells. Exp. Cell Res..

[195] Reipas K.M., Law J.H., Couto N., Islam S., Li Y., Li H., Cherkasov A., Jung K., Cheema A.S., Jones S.J. (2013). Luteolin is a novel p90 ribosomal S6 kinase (RSK) inhibitor that suppresses Notch4 signaling by blocking the activation of Y-box binding protein-1 (YB-1). Oncotarget.

[196] Li C., Wu H., Yang Y., Liu J., Chen Z. (2018). Sesquiterpene lactone 6-O-angeloylplenolin reverses vincristine resistance by inhibiting YB-1 nuclear translocation in colon carcinoma cells. Oncol. Lett..

[197] Gunasekaran V.P., Nishi K., Sivakumar D., Sivaraman T., Mathan G. (2018). Identification of 2,4-dihydroxy-5-pyrimidinyl imidothiocarbomate as a novel inhibitor to Y box binding protein-1 (YB-1) and its therapeutic actions against breast cancer. Eur. J. Pharm. Sci..

[198] Ma J.W., Hung C.M., Lin Y.C., Ho C.T., Kao J.Y., Way T.D. (2016). Aloe-emodin inhibits HER-2 expression through the downregulation of Y-box binding protein-1 in HER-2-overexpressing human breast cancer cells. Oncotarget.

[199] Tanaka T., Saito H., Miyairi S., Kobayashi S. (2021). 7-Hydorxyindirubin is capable of specifically inhibiting anticancer drug-induced YB-1 nuclear translocation without showing cytotoxicity in HepG2 hepatocellular carcinoma cells. Biochem. Biophys. Res. Commun..

[200] Wang J., Gibbert L., Djudjaj S., Alidousty C., Rauen T., Kunter U., Rembiak A., Enders D., Jankowski V., Braun G.S. (2016). Therapeutic nuclear shuttling of YB-1 reduces renal damage and fibrosis. Kidney Int..

[201] Higashi K., Tomigahara Y., Shiraki H., Miyata K., Mikami T., Kimura T., Moro T., Inagaki Y., Kaneko H. (2011). A novel small compound that promotes nuclear translocation of YB-1 ameliorates experimental hepatic fibrosis in mice. J. Biol. Chem..

[202] Houles T., Roux P.P. (2018). Defining the role of the RSK isoforms in cancer. Semin. Cancer Biol..

